# Multiomics and Systematic Analyses Reveal the Roles of Hemoglobin and the HIF‐1 Pathway in Polycystic Ovary Syndrome

**DOI:** 10.1002/advs.202411679

**Published:** 2025-02-14

**Authors:** Guiquan Wang, Weian Mao, Yurong Zhang, Haiyan Yang, Ming Zhu, Yan Li, Wei Chen, Yi Chen, Chen Lou, Ping Li, Hsun‐Ming Chang, Shuai Yuan, Yue Zhao, Liangshan Mu

**Affiliations:** ^1^ Department of Reproductive Medicine Women and Children's Hospital School of Medicine Xiamen University Xiamen 361003 China; ^2^ Xiamen Key Laboratory of Reproduction and Genetics Xiamen 361023 China; ^3^ Department of Obstetrics and Gynecology The Second Affiliated Hospital of Wenzhou Medical University Wenzhou 325027 China; ^4^ State Key Laboratory of Female Fertility Promotion Center for Reproductive Medicine Department of Obstetrics and Gynecology Peking University Third Hospital National Clinical Research Center for Obstetrics and Gynecology (Peking University Third Hospital) Key Laboratory of Assisted Reproduction Ministry of Education Beijing Key Laboratory of Reproductive Endocrinology and Assisted Reproductive Technology Beijing 100191 China; ^5^ Reproductive Medicine Center The First Affiliated Hospital of Wenzhou Medical University Wenzhou 325000 China; ^6^ The First School of Medicine Wenzhou Medical University Wenzhou 325035 China; ^7^ Reproductive Medicine Center Zhongshan Hospital Fudan University Shanghai 200032 China; ^8^ Department of Obstetrics and Gynecology China Medical University Hospital Taichung 40400 Taiwan; ^9^ Unit of Cardiovascular and Nutritional Epidemiology Institute of Environmental Medicine Karolinska Institute Stockholm 17177 Sweden

**Keywords:** causality, hemoglobin, hyperandrogenism, hypoxia‐inducible factor, polycystic ovary syndrome

## Abstract

Polycystic ovary syndrome (PCOS) affects reproductive and cardiometabolic health, yet its pathogenesis remains unclear. Emerging evidence links hemoglobin levels to metabolic disorders, suggesting a potential role in PCOS development. Here, we integrated a large‐scale cohort study, Mendelian randomization (A genetic tool to infer causal relationships), bioinformatics analyses, and in vitro experiments to investigate the relationship between hemoglobin levels and PCOS. In a cohort of 20 602 women, each 10 g L^−1^ elevation in hemoglobin levels is associated with 22% higher odds of PCOS (adjusted odds ratio: 1.22, 95% confidence interval: 1.15–1.29, *P* < 0.001) and PCOS manifestations, particularly hyperandrogenism. Mendelian randomization analysis confirms that higher hemoglobin levels are associated with increased PCOS risk and elevated testosterone levels. The hypoxia‐inducible factor 1 (HIF‐1) pathway is enriched, identifying three testosterone‐associated genes (nuclear factor kappa B *(NFKB1)*, insulin receptor *(INSR)*, protein kinase C alpha. Colocalization and druggability analysis supports shared genetic regions and confirmed these genes as druggable targets. Upregulation of *NFKB1* and *INSR* are confirmed in both blood and ovarian granulosa cells of PCOS patients. The findings demonstrate that higher‐end normal hemoglobin levels are associated with increased PCOS risk, potentially through a mechanism of elevating testosterone levels involving the HIF‐1 pathway.

## Graphical abstract

High‐end normal hemoglobin levels are associated with an increased odds of polycystic ovary syndrome (PCOS), potentially through a mechanism of elevating testosterone levels involving the hypoxia‐inducible factor 1 (HIF‐1) pathway. These findings provide novel insights into the pathophysiology of PCOS and highlight that hemoglobin levels may serve as a translationally relevant target for understanding and management of PCOS.

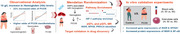

## 1 Introduction

Polycystic ovary syndrome (PCOS) is one of the most common reproductive and endocrine disorders in women of reproductive age, with a global prevalence ranging from 5% to 18%.^[^ ^1^ ^]^ It is characterized by three typical features: hyperandrogenism (clinical or biochemical), ovulation dysfunction (oligo‐ovulation or anovulation), and polycystic ovarian morphology (PCOM).^[^ ^2^ ^]^ The presence of PCOS has a range of implications for women's health. In addition to being one of the main causes of infertility disorders, it can also increase the risk of other health problems, such as type 2 diabetes, metabolic syndrome, and cardiovascular disease.^[^ ^3^, ^4^ ^]^ Despite the high prevalence and lifelong consequences, the etiology and pathophysiology of PCOS remain largely obscure.

Hemoglobin (Hb) plays an important role in the transport and storage of oxygen and its levels are regulated by genetic and environmental factors and vary according to age, gender, race, and living altitude.^[^ ^5^ ^]^ It is generally accepted that high‐end Hb levels within the normal range are beneficial to health.^[^ ^6^ ^]^ However, increasing evidence suggests that higher Hb levels are associated with adverse health effects.^[^ ^7^ ^]^ A few previous studies have reported associations between Hb levels and insulin resistance, hypertension, dyslipidemia, and metabolic syndrome.^[^ ^8^, ^9^, ^10^ ^]^ Although several small‐scale observational studies (each with fewer than 100 PCOS patients) have explored the relationship between Hb levels and PCOS, the findings have been inconsistent.^[^ ^11^, ^12^ ^]^ The potential for biased results and misinterpreted statistical significance might also arise from inadequate adjustment for confounding factors, as these studies considered only age and body mass index (BMI) as confounders, respectively. Moreover, neither the causal association between Hb levels and the onset and progression of PCOS nor the underlying mechanisms have yet been explored.

Mendelian randomization (MR) is a widely used epidemiological approach, employing genetic variants as instrumental variables (IVs) for exposure to draw causal inferences.^[^ ^13^ ^]^ By minimizing residual confounding and reverse causation, MR analyses are more likely to offer causal estimations compared to traditional observational approaches. Emerging studies have used MR to identify causal associations between PCOS and various factors, but the causal analysis with Hb has not been reported.^[^ ^14^ ^]^ Moreover, the integration of MR with pathway enrichment analysis can provide novel insights into the etiology and molecular mechanisms of diseases. Furthermore, leveraging genomics within MR offers a cost‐effective means to identify potential therapeutic targets in the early stages of drug discovery.^[^ ^15^ ^]^ Through the integration of molecular quantitative trait locus, such as gene expression quantitative trait loci (eQTL) or protein quantitative trait loci (pQTL), with genome‐wide association (GWAS) studies, target genes linked to risk variants can be discerned via causal inference.^[^ ^16^ ^]^ Colocalization analysis could further identify these targets, highlighting genes with robust genetic associations and thus higher therapeutic potential.^[^ ^17^ ^]^

In the present study, we designed a systematic research framework to investigate whether Hb levels contribute to PCOS. We first performed an observational analysis to examine the association between Hb concentration within the normal range and PCOS. We then conducted MR analyses to assess the causality and pathway enrichment analyses to reveal potential mechanistic pathways involved, supplemented by colocalization analysis and druggability assessment to detect prioritize potential therapeutic targets. Lastly, we validated the expressions of the identified key genes through in vitro experiments.

## 2 Results

### 2.1 Associations of Hb with PCOS and PCOS Manifestations in the Observational Study

Among the 20 602 women (median [interquartile range, IQR] age, 31^[^ ^28^, ^29^, ^30^, ^31^, ^32^, ^33^, ^34^, ^35^ ^]^ years) included in the retrospective cohort study, the median duration of infertility was 3 years (IQR 2–5), with 69.2% experiencing infertility due to tubal factor, 9.5% of diagnosed with endometriosis, and 6.6% had a history of ovarian surgery. A total of 3732 (18.1%) were diagnosed with PCOS according to the Rotterdam criteria; 964 (4.7%), 412 (2.0%), 511 (2.5%), and 1845 (9.0%) were diagnosed with PCOS phenotypes A, B, C, and D, respectively, according to the categorization by the National Institutes of Health (NIH); 5198 (25.2%), 5650 (27.4%), and 3215 (15.6%) presented ovulatory dysfunction, PCOM, and hyperandrogenism, respectively. Table S1 in the Supporting Information summarizes the baseline characteristics overall and across the tertiles of Hb concentrations. In general, women with higher Hb concentrations were more likely to present PCOS, all PCOS phenotypes and all three separate manifestations of PCOS.

**Figure 1:**
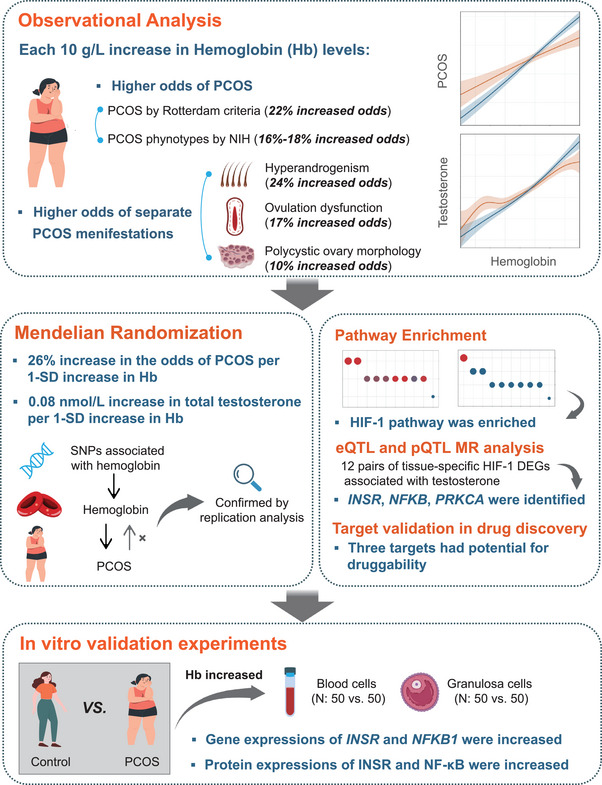
Central illustration of this study. Systematic analyses of the association between hemoglobin and polycystic ovary syndrome identify the potential effect of HIF‐1 pathway. Abbreviations: DEGs, differentially expressed genes; eQTL, expression quantitative trait loci; HIF, hypoxia‐inducible factor pathway; INSR, insulin receptor; NFKB, nuclear factor kappa‐light‐chain‐enhancer of activated B cells; PCOS, polycystic ovary syndrome; PRKCA, protein kinase C alpha; SD, standard deviation; SNP, single nucleotide polymorphism.

**Figure 2:**
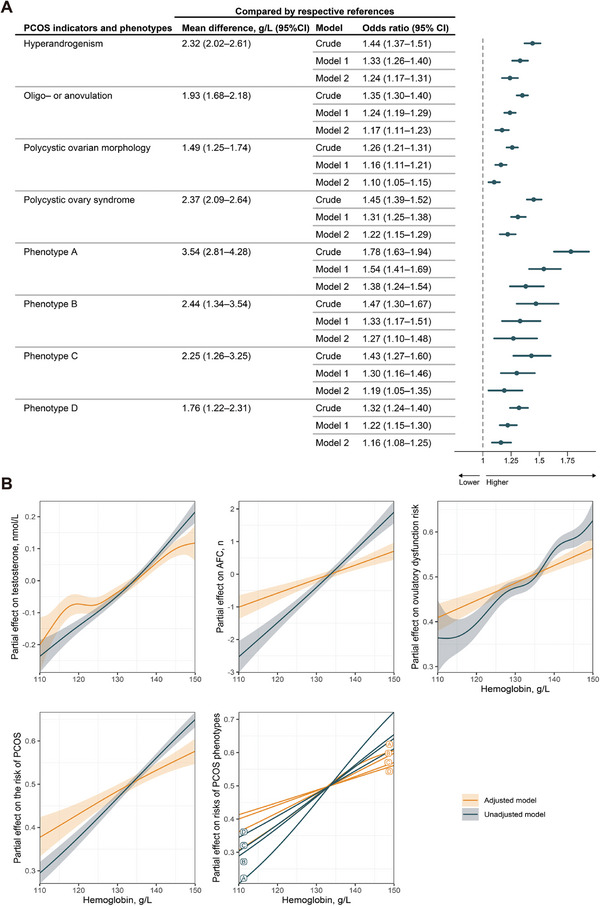
The associations between Hb levels and PCOS and its manifestations. A) Univariate and multivariate logistic regression model analysis to characterize the associations between Hb levels and PCOS, PCOS phenotypes, and separate manifestations. Model 1 was adjusted for intervals of appointment date, age, body mass index, and education; Model 2 was additionally adjusted for systolic blood pressure, endometriosis status, history of ovary surgery, LH/FSH ratio, fasting blood glucose, total cholesterol, triglycerides, and high‐ and low‐density lipoprotein levels. B) Nonlinear associations between Hb levels and PCOS, PCOS phenotypes, and separate manifestations, adjusted for confounders in Model 2. Abbreviations: PCOS, polycystic ovary syndrome; LH, luteinizing hormone; FSH, follicle‐stimulating hormone.

Logistic regressions demonstrated that elevated Hb levels within the normal range were associated with increased odds of all PCOS phenotypes and individual clinical manifestations of PCOS (**Figures** **1** and **2**A). We observed higher Hb levels in PCOS patients according to the Rotterdam criteria compared with non‐PCOS (mean difference 95% confidence interval [CI]: 2.37 [2.09–2.64] g L^−1^). After adjustments for most confounders, for every 10 g L^−1^ increase in Hb levels, there was a 22% increase in the odds of developing PCOS (adjusted odds ratio [aOR] [95% CI]: 1.22 [1.15–1.29]). As Hb levels increased, the odds of hyperandrogenemia increased the most significantly (aOR [95% CI], 1.24 [1.17–1.31]). Among the four PCOS phenotypes, the greatest increase in the odds was observed in phenotype A (aOR [95% CI], 1.38 [1.24–1.54]), in which all three clinical manifestations occurred simultaneously. Generalized additive model (GAM) partial effect curves confirmed the positive associations between Hb levels and the clinical outcomes (Figure 2B). There was a safe plateau phase in the association with testosterone levels when Hb ranged from 120 to 125 g L^−1^, while associations with antral follicle count (AFC), ovulatory dysfunction, PCOS and PCOS phenotypes roughly remained linearity in the adjusted models.

### 2.2 MR Analyses Revealed the Causal Association of Hb Levels with PCOS and Testosterone

**Figure 3:**
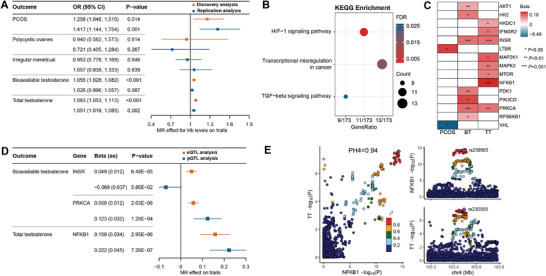
MR results and pathway enrichment analysis. A) Associations of Hb levels with PCOS and related traits according to the Mendelian randomization analysis. B) Significant results for KEGG pathway enrichment analysis of candidate genes from Hb IVs used in MR analysis. C) Significant tissue‐specific eQTL MR results in whole blood and ovary. D) eQTL and pQTL MR results for INSR, NFKB1, and PRKCA on testosterone levels. E) LocusCompare plots comparing genetic signals for NFKB1 levels and TT levels in colocalization analysis. Abbreviations: BT, bioavailable testosterone; TT, total testosterone; SHBG, sex hormone binding globulin; PH4, posterior probability for shared causal variants.

The *F*‐statistics for IVs in all MR analyses exceeded 10, suggesting that the MR estimates were unlikely to be subject to weak instrument bias. In the discovery stage, our MR analysis demonstrated that genetically predicted elevated Hb levels, per 1‐ standard deviation (SD) increase, were associated with increased odds of PCOS according to the inverse‐variance weighted (IVW) method (odds ratio [OR] [95% CI]: 1.26 [1.05–1.51], *P* = 0.014), along with increased bioavailable testosterone (BT) levels (*β* [standard error, SE]: 0.05 [0.01] nmol L^−1^, *P* = 6.110 × 10^−5^), and higher total testosterone (TT) levels (*β* [SE]: 0.08 [0.01] nmol L^−1^, *P* = 1.348 × 10^−8^) (Figure 1 and **Figure** **3**A).

Subsequent replication analysis using another GWAS meta‐analysis statistics of Hb levels, with a smaller sample size, confirmed significant associations with PCOS risk and TT levels. The analysis indicated associations between increased Hb levels and higher risk of PCOS (OR 1.42 [95% CI]: [1.14–1.75], *P* = 0.001) and elevated TT levels (*β* [SE]: 0.05 [0.02] nmol L^−1^, *P* = 0.002) (Figure 3A). Other MR methods provided statistically consistent estimates of effect directions (Table S2, Supporting Information). The type 1 error rate caused by sample overlap between Hb and PCOS‐related phenotypes was controlled under 0.05, suggesting that the substantial effect of partial overlap on causality was acceptable (Table S3, Supporting Information).

In the reverse direction of MR analyses, no significant association was found between genetic susceptibility to PCOS and Hb levels in either the discovery or replication analysis (Table S4, Supporting Information). The multivariate Mendelian randomization (MVMR) analysis revealed that the association between genetically predicted Hb levels and increased odds of PCOS remained significant, even after adjusting for the influences of BT (aOR [95% CI]: 1.25 [1.05–1.49], *P* = 0.014) and TT (aOR [95% CI]: 1.23 [1.03–1.48], *P *= 0.025), respectively. Similar significant associations were observed in the replication analysis of Hb levels in PCOS patients after adjustment for BT (aOR [95% CI]: 1.36 [1.20–1.68], *P* = 0.004) and TT (aOR [95% CI]: 1.29 [1.01–1.65], *P *= 0.039) (Table S5, Supporting Information).

### 2.3 Pathway Enrichment Identified the Hypoxia‐Inducible Factor 1 (HIF‐1) Pathway

Employing Hb‐associated genetic variants derived from the largest GWAS study, three pathways, including HIF‐1 signaling pathway, transcriptional misregulation in cancer, and TGF‐beta signaling pathway, were identified via Kyoto Encyclopedia of Genes and Genomes (KEGG) pathway enrichment analysis, with a false discovery rate (FDR) less than 0.05 as the significance threshold (Figure 3B and Tables S6 and S7, Supporting Information).

### 2.4 Key Genes Were Identified Using eQTL and pQTL MR Analyses

Using eQTL data of genes within the HIF‐1 pathway sourced from the Genotype‐Tissue Expression (GTEx) v8 database across various tissues including whole blood, ovarian, hypothalamic, and pituitary tissues, we identified three pairs of genetically proxied HIF‐1 pathway genes significantly associated with testosterone levels. Employing a Bonferroni‐corrected threshold of *P* < 4.9 × 10^−4^ (Bonferroni‐corrected threshold of 0.05 for 102 HIF‐1 pathway eQTLs as exposures) for multiple testing, these associations were found between specific tissue expressions of INSR (insulin receptor), NFKB1 (nuclear factor kappa B), and PRKCA (protein kinase C alpha), and genetically proxied testosterone levels (Figures 1 and 3C,D and Table S8, Supporting Information). Consequently, our subsequent in vitro experiments focused on validating these three genes.

Significant associations in the eQTL MR analysis were further replicated using pQTL for PRKCA (*β* [SE]: 0.12 [0.03] nmol L^−1^, *P* = 1.276 × 10^−4^) and NFKB1 (*β* [SE]: 0.22 [0.05] nmol L^−1^, *P* = 7.263 × 10^−7^) (Figure 3D). Moreover, we detected strong colocalization evidence for NFKB1 and TT (posterior probability for hypothesis [PH] 4 = 0.94), indicating a common variant shared between NFKB1 and increased TT levels (Figure 3E and Table S9, Supporting Information).

### 2.5 Druggability Assessment Suggested Potential Development Prospects

To assess potential development prospects, we collected data on clinical trials from drug databases for the three targets identified in the MR analysis to evaluate their druggability. Our findings indicate that these three proteins were considered druggable targets albeit at varying stages of drug discovery. Clinical trials predominantly focus on medications aimed at treating insulin resistance, as evidenced by the available data (Table S10, Supporting Information).

### 2.6 In Vitro Experiments Confirmed the Expressions of the Identified Key Genes

**Figure 4:**
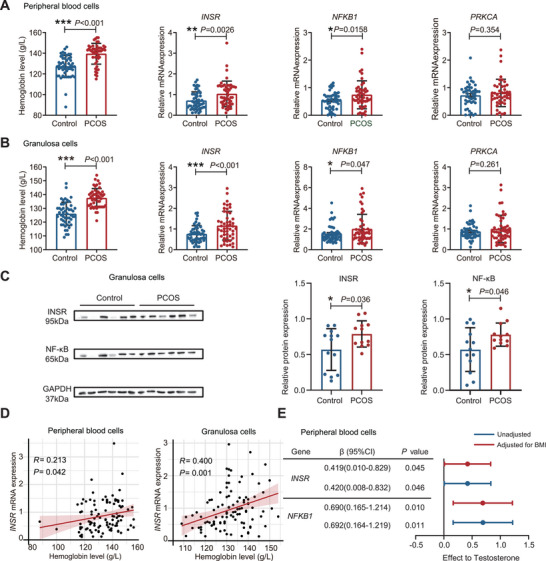
In vitro validation experiments in peripheral blood cells and granulosa cells. Hb levels, qPCR analyses of *INSR*, *NFKB*1 and *PRKCA* expression in A) peripheral blood cells and B) granulosa cells. The blue dots indicate the mRNA levels in the control group, and the red dots indicate the mRNA levels in the PCOS patients. *P* values, two‐tailed Student's *t*‐test or Mann‐Whitney test. C) Representative Western blot analysis of INSR and NF‐κB in the granulosa cells of the control and PCOS groups in three independent experiments, with the GAPDH level used as a control. D) Linear regression analysis of hemoglobin levels and *INSR* and *NFKB1* gene expression in peripheral blood cells and granulosa cells after adjusting for BMI. E) Linear regression analysis of *INSR* and *NFKB1* gene expression and testosterone level in peripheral blood cells before and after adjusting for BMI.

The transcription levels of the identified genes, *INSR*, *NFKB1*, and *PRKCA*, were examined in both the peripheral blood cells and ovarian granulosa cells of women with PCOS and controls. Hb levels were significantly greater in PCOS patients than in controls. The gene expression levels of *INSR* and *NFKB1* were significantly increased in both peripheral blood cells and granulosa cells of women with PCOS than in those of control individuals, while no significant difference was observed in the *PRKCA* transcription level between the two groups (Figure 1 and **Figure** **4**A,B). The protein expression levels of INSR and NF‐κB were also elevated in the granulosa cells of PCOS patients compared with those in the controls (Figure 4C). Detailed clinical information of the PCOS and control groups was indicated in Tables S11 and S12 in the Supporting Information. In addition, linear regression analysis indicated a positive correlation between Hb levels and *INSR* expression in both peripheral blood cells and granulosa cells after adjusting for BMI (Figure 4D and Table S13, Supporting Information). Moreover, the expressions of *INSR* and *NFKB1* in peripheral blood cells showed a positive correlation to testosterone levels, with or without adjustment for BMI (Figure 4E).

## 3 Discussion

In the present study, we conducted a systematic investigation into the association between Hb levels and PCOS. We found that elevated Hb levels, even within the normal range, were associated with increased odds of PCOS and its manifestations. Our MR analyses provided robust evidence supporting a causal association between Hb levels and both PCOS and testosterone levels. Notably, the Hb‐associated genetic variants were significantly enriched in the HIF‐1 pathway, and two key genes associated with testosterone levels, *INSR* and *NFKB1*, were both confirmed to be upregulated in PCOS patients. Colocalization analysis suggested that NFKB1 shares a causal variant with TT levels, highlighting its potential as a therapeutic target for the treatment of hyperandrogenic states in PCOS patients. These findings provided new insights for the molecular mechanisms underlying PCOS pathogenesis and potential therapeutic targets for clinical intervention.

Few previous studies have explored the association between Hb levels and PCOS. One study reported higher Hb levels in both lean and overweight/obese subgroups of adolescents with PCOS when compared to the age‐matched controls.^[^ ^12^ ^]^ However, some studies have reported no significant differences in hematologic values between PCOS patients and controls.^[^ ^11^ ^]^ In addition, Hb levels are also linked to a wide range of disorders, including metabolic syndrome, hyperuricemia, and nonalcoholic fatty liver disease, suggesting its extensive involvement in metabolic regulation.^[^ ^18^, ^19^, ^20^ ^]^ The underlying mechanisms of these associations have not yet been thoroughly elucidated, but insights from previous studies may provide some potential evidence. In our observational analysis, elevated Hb levels were significantly associated with the risk of PCOS as well as its inherent indicators, such as hyperandrogenemia, ovulatory dysfunction, and polycystic ovarian morphology. The observed 22% increased odds of PCOS per 10 g L^−1^ rise in hemoglobin levels suggest that even high‐normal Hb levels may serve as a significant biomarker for heightened PCOS risk. Given the multifactorial nature and high prevalence of PCOS, these findings highlight the potential utility of Hb levels in risk stratification and early identification of individuals at greater risk for PCOS. Hb is essential for oxygen transport in the body and provides the required oxygen for cellular metabolism, energy production, and biological functions.^[^ ^21^ ^]^ Elevated Hb levels may indicate a state of chronic hypoxia or anoxia, which has been shown to lead to oxidative stress, insulin resistance, and inflammation.^[^ ^22^, ^23^ ^]^ All of these factors are associated with the onset and progression of PCOS. The relationship between high Hb concentrations and increased blood viscosity, along with alterations in plasma volume, endothelial cell dysfunction, or changes in the ovarian microenvironment, may also explain this association. Elevated Hb levels can lead to insulin resistance by increasing blood viscosity, reducing blood flow, and influencing the supply of oxygen and metabolites to the tissue.^[^ ^24^ ^]^ Furthermore, an alternative function of Hb may be as a carrier of nitric oxide (NO), which can modulate vascular endothelial function via the arginine‐NO pathway.^[^ ^25^, ^26^ ^]^ Consequently, higher Hb levels may induce endothelial dysfunction, also leading to insulin resistance. Moreover, alterations in Hb levels or function could impact oxygen delivery to the ovarian microenvironment, potentially leading to changes in ovarian autophagy, oxidative stress, and mediating the signaling that drives luteinization.^[^ ^27^, ^28^, ^29^, ^30^ ^]^

We found increased Hb levels in women with PCOS compared to controls, and the single nucleotide polymorphisms (SNPs) and candidate genes closely related to Hb levels were mainly enriched in the HIF‐1 signaling pathway. A previous study showed that higher Hb levels were related to metabolic disorders and the inhibition of the HIF‐1 pathway.^[^ ^7^ ^]^ However, there are still inconsistent conclusions regarding the changes in the HIF‐1 pathway in PCOS patients. Studies have shown that the expression of HIF‐1α messenger RNA (mRNA and protein in the granulosa cells of PCOS patients and in the ovaries of PCOS‐like rats was decreased.^[^ ^31^, ^32^, ^33^ ^]^ However, the HIF‐1α protein level in granulosa cells was also not significantly different between control and PCOS patients.^[^ ^31^ ^]^ Moreover, HIF prolyl hydroxylase activity was increased, which might lead to the inhibition of the HIF‐1α signaling pathway.^[^ ^34^ ^]^ There was also inconsistent result showing that the mRNA and protein levels of HIF‐1α were upregulated and induced by reactive oxygen species.^[^ ^35^ ^]^

Several studies have indicated a relationship between HIF1 and androgens, especially testosterone. Under hypoxia conditions, activated HIF1 was found to bind to the Star promoter region and repress the expression of StAR, thus impairing the synthesis of testosterone.^[^ ^36^ ^]^ Research has also suggested that the promoter of Hsd3b1, a gene involved in testosterone production, may be a potential target of HIF1α.^[^ ^37^ ^]^ Additionally, adverse environmental conditions, such as air pollution and microplastic contamination, may disrupt HIF1α signaling pathways, thereby impacting hormone synthesis via sirtuin 1 (SIRT1), extracellular regulated protein kinases 1/2 (ERK1/2), mitogen‐activated protein kinase (MAPK), or protein kinase B (AKT) pathways.^[^ ^38^, ^39^ ^]^ However, current research has predominantly focused on males and further studies are needed to explore this relationship in females to elucidate the role of HIF in hormone synthesis and to explore how dysregulation of the HIF1 signaling pathway contributes to the pathogenesis of diseases. HIF1α also affects women's reproductive function by regulating the development and excretion of follicles.^[^ ^40^ ^]^ The luteinization of GCs during early corpus luteum formation was found to be regulated by HIF1α‐BNIP3 mediated through autophagy.^[^ ^41^ ^]^ HIF‐1α was found to bind to histone 3/4‐acetylated androgen receptor (AR) promoter and transcriptionally activate AR via forming a transcriptional activation complex with BRD4, then promote ovarian fibrosis in PCOS‐like rat.^[^ ^42^ ^]^ Furthermore, elevated palmitic acid levels in obese women could cause the collagen‐crosslinking enzyme lysyl oxidase dysregulation via activation of HIF‐1α, resulting in abnormal collagen deposition in the ovary and anovulation.^[^ ^43^ ^]^ In addition, activation of the HIF pathway is known to alleviate metabolic defects including impaired glucose tolerance, elevated fasting glucose levels and insulin resistance in PCOS mouse model.^[^ ^44^ ^]^ To summarize, HIF1 pathway may contribute to pathogenesis of PCOS by modulating hormone synthesis, ovulation, and metabolic processes, and the underlying mechanisms warrant further in‐depth exploration.

Our MR analysis and pathway enrichment analysis identified genes related to the HIF‐1 pathway, with the expression levels of INSR and NFKB1 significantly positively correlated with testosterone levels. Previous GWAS have demonstrated associations between *INSR* variants and PCOS in Chinese and European individuals populations, suggesting a potential impact on metabolic disruption, such as insulin resistance and obesity, in PCOS patients.^[^ ^45^, ^46^ ^]^ In the granulosa cells of PCOS patients, the *INSR* mRNA expression was significantly lower than that in the control group.^[^ ^47^ ^]^ There were also some findings that *INSR* mRNA showed no significant difference in the granulosa cells of PCOS patients or in the ovaries of PCOS‐like mice.^[^ ^48^, ^49^ ^]^ These findings were not in line with our aforementioned higher level of *INSR*. A previous study showed that insulin sensitivity is decreased in PCOS granulosa cells, impairing the glucose uptake process.^[^ ^49^ ^]^ The elevated expression of *INSR* in the present study might be an adaptive compensatory effect. HIF‐1α can be induced by the activation of receptor tyrosine kinases. To our knowledge, the relationship between *INSR*, a member of the receptor tyrosine kinases (RTK) family, and HIF‐1α in PCOS has not been reported, which requires further exploration.

Inflammation and hyperandrogenism have complex causes and effects on each other, and hyperandrogenism can aggravate inflammation, in which NF‐κB is a main regulator and orchestrates different steps.^[^ ^50^ ^]^ We detected enhanced expression of *NFKB1* in the granulosa cells of PCOS patients, which was consistent with the chronic inflammatory status in PCOS. Previous studies have also indicated the upregulated expression or the activation of NF‐κB in women with PCOS.^[^ ^51^ ^]^ A crosstalk between NF‐κB and HIF has been associated with several pathological conditions. HIF‐1α can regulate NF‐κB through an evolutionarily conserved negative feedback mechanism, and NF‐κB has been shown to act as a direct modulator of HIF‐1α expression in inflammation and hypoxia.^[^ ^52^ ^]^ Colocalization analysis indicated that *NFKB1* shares same causal genetic variants with TT levels, suggesting that targeting this way has the potential to modulate the hyperandrogenemia state of PCOS. Of the existing drug development targeting *NFKB1*, two major compounds, HE3286 and Triflusal, are in clinical trials, but there are no studies related to testosterone levels. In the hypoxia‐reoxygenation rat models, Triflusal showed neuroprotection by regulating oxidative stress, suggesting that the drug may be involved in the metabolic response to hypoxia, but whether it is through the HIF‐1 pathway needs to be further explored.^[^ ^53^ ^]^

This study has several strengths. We systematically investigated, for the first time, the nature of the associations between Hb levels and PCOS, as well as PCOS‐related traits. Hb levels were positively associated with the odds of PCOS and testosterone levels, and the genetic causal associations were rigorously confirmed step by step. Our findings unveiled the involvement of the HIF‐1 pathway in this process, shedding light on key genes such as *INSR* and *NFKB1*. The differential expression of these genes and their association with Hb levels in PCOS patients were further confirmed using in vitro experiments. In addition, while the testosterone level has been identified as a type of hematopoietic hormone that might confound these associations, we conducted multivariable MR analysis to adjust for the potential effects of androgens on the association with PCOS.^[^ ^54^ ^]^ Compared to traditional observational studies, MR offers a unique advantage by elucidating the causal effects of specific exposures on outcome traits, minimizing confounding and reverse causality.

## 4 Limitation

The limitations of our study include the observational nature of human data and that we did not quantify serum iron or ferritin levels, which are both closely related to Hb levels. To address this, we included only individuals with normal Hb levels. Although we have also excluded conditions known to affect hemoglobin levels (see the Experimental Section) and adjusted for BMI, blood pressure, fasting blood glucose, and lipid profiles, the residual influence of unmeasured lifestyle factors (e.g., diet, physical activity, smoking, and alcohol consumption) cannot be excluded due to the nature of our retrospective data. Future studies incorporating detailed lifestyle data are warranted to more comprehensively evaluate these potential influences. Another limitation is that the study population was included from a reproductive center, which may limit the generalizability of our findings to broader populations, especially those without infertility concerns. Therefore, epidemiological research involving a larger, more representative sample of the general population is still needed to confirm our findings. In addition, the MR analyses were restricted to individuals of European ancestry. Although the associations of Hb levels with PCOS and related traits were identified in the Han Chinese population, whether the results obtained can be extended to other ethnicities should be determined with caution.

## 5 Conclusion 

In conclusion, our comprehensive study unveils a potential link between elevated Hb levels and an increased risk of PCOS and its individual traits, particularly hyperandrogenemia. Moreover, we identify the HIF‐1 pathway as a key regulatory mechanism in this association. These findings indicate that low‐end Hb levels may be favorable for PCOS patients and may be potentially beneficial for the prevention of PCOS. Additionally, our results imply that hypoxia, or reduced oxygen levels, may hold potential as a preventive measure for PCOS. Further exploration of these pathways and their interactions may offer novel insights into the pathogenesis of PCOS and potential therapeutic strategies.

## 6 Experimental Section

### Source of Data

For the observational study, 24 757 women underwent reproductive examinations and laboratory testing at the First Affiliated Hospital of Wenzhou Medical University and finished their first in vitro fertilization (IVF) treatments due to infertility between January 2010 and December 2021. This center is located in the coastal plain region of East Asia, where over 95% of the individuals are local residents (the specific residential locations were not disclosed due to patient privacy concerns). While a small number of individuals originate from areas outside the plain region, all have resided and worked in this area for an extended period. Among the 22 042 women with normal Hb concentrations (ranging from 110 to 150 g L^−1^), 1440 were excluded for chromosomal abnormalities, thyroid dysfunction, hyperprolactinemia (or pituitary microadenoma), current smoking status, kidney (or liver) dysfunction, history of hematological disorders or radiotherapy or chemotherapy (Figure S1, Supporting Information). All data were collected from the health information system customized for reproductive centers to store clinical, laboratory, demographic, and reproductive‐specific information. This study was approved by the institutional review committee of the hospital (Approval No. KY2022‐R096), waiving the need for informed consent due to deidentified data. This observational study was conducted in accordance with the Strengthening the Reporting of Observational Studies in Epidemiology (STROBE) statement.^[^ ^55^ ^]^

For the MR analyses, meta‐analysis data from the largest publicly available GWAS summary statistics were utilized for Hb concentrations in 563 085 participants of European descent for the discovery analysis. Genetic associations were adjusted for age, age^2^, sex, and top principal components.^[^ ^56^ ^]^ To examine the robustness of the association, summary statistics were used from another large‐scale Hb GWAS including 173 480 individuals of European ancestry in the replication analysis, which made adjustments including age, sex, and common factors known to be influential on Hb levels such as BMI, alcohol consumption, and smoking status.^[^ ^57^ ^]^ Regarding PCOS and related traits, corresponding summary‐level data were extracted from the hitherto largest GWASs.^[^ ^58^, ^59^, ^60^ ^]^ GWAS statistics for PCOS were derived from a meta‐analysis by Day F et al., which included a European population of 10 074 patients and 103 164 controls, adopting the NIH or Rotterdam diagnostic criteria for case definition.^[^ ^58^ ^]^ The dataset of tissue‐specific eQTL data of target genes were obtained from the GTEx web portal, which included 948 donors and 17 382 samples from 54 tissues.^[^ ^61^ ^]^ *Cis*‐pQTLs for the corresponding plasma proteins were extracted from the deCODE study where aptamers were measured using the SomaScan platform among 35 559 Icelanders.^[^ ^62^ ^]^ Details for all phenotypes included in MR analysis were taken from the published studies described in Table S14 in the Supporting Information.

For in vitro validation experiments, Chinese women with PCOS and healthy women as controls were recruited from the Reproductive Medicine Center of Peking University Third Hospital from December 2021 to May 2023. The diagnosis of PCOS was based on the Rotterdam criteria.^[^ ^63^ ^]^ Patients with other etiologies for hyperandrogenemia or ovulatory dysfunction (Cushing syndrome, 21‐hydroxylase deficiency, thyroid disease, androgen‐secreting tumors, congenital adrenal hyperplasia, and hyperprolactinemia) were excluded. The control subjects had regular menstrual cycles, normal ovarian morphology, and normal levels of hormones. None of the women used medications known to affect metabolic function or reproductive function within 3 months before enrollment. This part of the study was approved by the Ethics Committee of Peking University Third Hospital (No. 2021‐533‐01) and informed consent was obtained from all participants.

### Outcomes of Interest

The primary outcome was clinically defined PCOS according to the Rotterdam criteria.^[^ ^63^ ^]^ The secondary outcomes were PCOS phenotypes according to the NIH consensus panel,^[^ ^64^ ^]^ three indicators of PCOS, and corresponding continuous forms: hyperandrogenism (testosterone concentrations), PCOM (AFC), and ovulatory dysfunction. Hyperandrogenism was defined as a serum testosterone level > 2 nmol L^−1^ and ovulatory dysfunction was defined as infrequent menstrual bleeding (>38 d) or amenorrhea.^[^ ^65^, ^66^ ^]^ The testosterone levels and menstrual patterns were obtained under the condition that no hormonal treatments were used. AFC was defined as the total number of small follicles (with a diameter of 2–10 mm) in both ovaries counted by a senior or above attending reproductive physician on days 2–3 of the menstrual cycle.

### Measurements Involved in This Study

For the observational study, age, BMI, education, systolic (and diastolic) blood pressure, infertility type, endometriosis, history of ovarian surgery, basal sexual hormones (follicle‐stimulating hormone [FSH], luteinizing hormone [LH], LH/FSH ratio, and estradiol), fasting blood glucose, total cholesterol, triglyceride, high‐ and low‐density lipoprotein as potential covariates were included. Patient anthropometric data were collected by nurses with at least 3 years of nursing experience. After overnight fasting for at least 8 h, peripheral blood samples were collected on days 2–4 of a natural menstrual cycle or when amenorrhea for over 40 d with follicular diameter not exceeding 10 mm. Basal AFC was taken to be the total number of small follicles (diameter of 2–10 mm) in both ovaries. Infertility type was classified as primary and secondary determined by pregnancy history. Endometriosis was identified according to International Classification of Diseases (tenth revision) code N80. Sex hormones were analyzed using an ultrasensitive enzyme‐linked immunosorbent assay (Unicel Dxl 800, Beckman Coulter, Brea, CA). Serum fasting blood glucose, triglyceride, high‐ and low‐density lipoprotein were measured using an autoanalyzer (AU 5800, Beckman Coulter, Brea, CA). Serum Hb levels were measured no more than one year prior to the initiation of IVF treatment, using an automatic blood analyzer (Sysmex Corporation). The detection range of Hb was 0–250 g L^−1^. For the testing method of biochemical parameters, both intra‐assay and interassay variations were less than 10%.

For the in vitro experiments, as patients’ biosamples were collected from an independent hospital, measurements of FSH, LH, estradiol, total testosterone levels, and androstenedione were performed using a Siemens Immulite 2000 immunoassay system (Siemens Healthcare Diagnostics, USA). Ultrasensitive two‐site enzymelinked immunosorbent assay (Ansh Labs, USA) was used to measure anti‐Müllerian hormone (AMH). The sensitivity of the measurement of FSH, LH, estradiol, total testosterone levels, androstenedione, AMH were 0.1 U L^−1^, 0.05 U L^−1^, 15 ng L^−1^, 0.5 nmol L^−1^, 0.3 µg L^−1^, 23 pg mL^−1^, and intra‐assay variation were 5.57%, 6.00%, 7.63%, 10.23%, 10.53%, and <5%, respectively. The serum levels of fasting serum glucose were determined by chemiluminescence method using Immulite 1000 system (DPC, USA). Total cholesterol, triglyceride, low‐density lipoprotein cholesterol (LDL‐C), and high‐density lipoprotein cholesterol (HDL‐C) were measured by a dry slide enzymatic colorimetric assay (Johnson, USA). The detection ranges of LDL‐C, HDL‐C, and Hb levels were 0.03–11.80 mmol L^−1^, 0.05–3.90 mmol L^−1^, and 0–250 g L^−1^, respectively, and intra‐assay variations were <5%, <5%, and ≤1.5%, respectively. Blood cells were collected by centrifugation at 300 g for 10 min for the quantitative analyses of target genes.

All laboratory data were uniformly measured and reviewed by the laboratory department of these two tertiary hospitals. All original data was recorded in the hospital information systems, had never been modified by nonmedical personnel, and only existed within the local area network of both hospitals.

### Mendelian Randomization Analyses

In MR, genetic variants, randomly allocated at conception, are used as IVs to proxy the effect of the exposure (i.e., Hb levels in this study). To construct IVs, SNPs associated with Hb levels were selected first at the genome‐wide significance level (*P* < 5 × 10^−8^). Then linkage disequilibrium (i.e., genetic correlation matrix) of these SNPs based on 1000 Genomes European reference panel was estimated and independent variants (linkage disequilibrium *r* ^2^ < 0.001 in a clumping window of 10 000 kb) as the IVs for Hb were selected. Data were extracted on the IVs from the outcome GWAS data and they were harmonized based on effect and noneffect alleles for subsequent MR analysis. To assess the bias from weak instruments, *F* statistics were calculated to judge the strength of IVs, with a threshold above ten as the conventional rule.

To investigate the causal association of Hb levels with PCOS manifestations, MR approach was used to test the association of genetically predicted Hb levels with PCOS and its related traits, including irregular menstrual cycle, polycystic ovaries, BT, and TT. Considering the possible interplay between androgens and Hb levels, an MVMR analysis was employed to account for BT or TT levels in subsequent validation stages. This adjustment was crucial to ascertain whether the observed MR effects of Hb concentrations on PCOS remained independent of testosterone levels.^[^ ^67^ ^]^ In MVMR analysis, all GWAS‐associated SNPs for both traits in the analytical model were included to assess whether the effects of Hb concentrations on PCOS were independent of BT or TT. The MR part of the study followed the Strengthening the Reporting of Observational Studies in Epidemiology Using Mendelian Randomization (STROBE‐MR) guideline (Table S15, Supporting Information).

### Pathway Enrichment Analyses and Pathway‐Specific Mendelian Randomization Analysis

Pathway enrichment analysis was conducted to provide insights into biological pathways. The gene annotation corresponding to IV was implemented in the Ensembl genome browser and then the gene symbol was mapped to the Entrez gene identifier.^[^ ^68^ ^]^ The KEGG database was used for pathway definition.^[^ ^69^ ^]^

As follow‐up analyses, MR analysis was conducted using eQTLs of pathway candidate genes related to PCOS and related traits. For all genes of the HIF‐1 pathway, eQTL data were extracted from the GTEx v8 database in whole blood, ovary, hypothalamus, and pituitary tissue, as Hb is present in whole blood tissue, and ovary, hypothalamus and pituitary tissue are the most relevant to the development of PCOS. The corresponding *cis*‐eQTLs were extracted according to the following criteria: variants within ± 1 Mb of each gene transcription start site, with *P* < 5 × 10^−4^ and *r* ^2^ < 0.1.^[^ ^61^ ^]^

*cis*‐pQTLs associated with plasma protein levels (*P* < 5 × 10^−8^) were also used as IVs to perform a validation analysis of eQTL MR results. *Cis*‐pQTLs were extracted from the deCODE Health study, where plasma aptamers were measured by the SomaScan platform among 35 559 Icelanders.^[^ ^62^ ^]^ *Cis*‐SNPs were defined as SNPs within 1 Mb from the gene encoding the protein, and SNPs with high linkage disequilibrium (*r* ^2^ > 0.01) were pruned. Since the number of SNPs available for colocalization in the eQTL study was limited, association analyses on pQTLs and testosterone levels were mainly performed. Lastly, to evaluate the druggability of proteins identified, the DrugBank, Dependency Map, and ChEMBL databases were searched to summarize the current stages of drug development targeting these proteins.

### Granulosa Cell Isolation

The individuals received a standard in vitro fertilization antagonist stimulation protocol and underwent transvaginal ultrasound‐guided follicle aspiration 36 h after human chorionic gonadotropin (hCG) administration. Mural granulosa cells were isolated from the follicular fluids aspirated during oocyte retrieval by Ficoll density gradient centrifugation as previously described.^[^ ^70^ ^]^

### RNA Extraction and Real‐Time Quantitative Polymerase Chain Reaction (qPCR) Analysis

Total RNA was extracted from peripheral blood cells and granulosa cells with TRIzol (Thermo Fisher Scientific, USA). RNA purity was determined by the absorption ratio (260/280 nm), which was 1.8–2.0 for all samples. One microgram of purified RNA was used to generate cDNA with a ReverTra Ace qPCR RT Kit (Takara, Dalian, China). Real‐time qPCR analysis was performed using SYBR Green PCR master mix (Invitrogen) and the QuantStudio 3 real‐time PCR system (Thermo Fisher Scientific). The qPCR primers used in this study were as follows: 5′‐TGGCGATGTTGGGAATGTG‐3′ and 5′‐GATGCGATAGCCCGTGAAGT‐3′ for *INSR*; 5′‐GCGGTGACAGGAGACGTGAA‐3′ and 5′‐CAAGTTGAGAATGAAGGTGGATG‐3′ for *NFKB1*; and 5′‐GCCTATGGCGTCCTGTTGTAT‐3′ and 5′‐GCTTGGCTGGGTGTTTGGT‐3′ for *PRKCA*.

### Western Blotting

Protein was extracted from granulosa cells using radio immunoprecipitation assay (RIPA) buffer supplemented with protease inhibitors and phosphatase inhibitors and quantified by a bicinchoninic acid (BCA) assay (BCA Protein Assay Kit; Pierce Thermo Scientific). Total protein extracts from granulosa cells were separated via 12% sodium dodecyl sulfate ‐ polyacrylamide gel electrophoresis (SDS‒PAGE). Western blotting was performed as described previously.^[^ ^61^ ^]^ The following primary antibodies were used according to the provided recommendations: anti‐insulin receptor beta (1:1000, ab69508, Abcam), anti‐NF‐κB (1:1000, 8242, Cell Signaling Technology), and anti‐GAPDH (glyceraldehyde‐3‐phosphate dehydrogenase) (1:1000, 5174, Cell Signaling Technology).

### Statistical Analysis—The Observational Study

Categorical variables were reported as number (percentage) and compared among Hb tertile groups using the Chi‐squared test.^[^ ^71^ ^]^ Numerical variables were reported as median (IQR) and compared using the Kruskall‐Wallis test.^[^ ^72^ ^]^ No imputation was performed for missing data. The associations of Hb concentrations with PCOS, PCOS phenotypes, hyperandrogenism, ovulatory dysfunction, and PCOM were tested first using logistic regression models. These models were fitted respectively adjusting for increasing numbers of covariates and the aOR was calculated: Model 1 adjusted for intervals of appointment date, age, BMI, and education; Model 2 additionally adjusted for systolic blood pressure, endometriosis, history of ovary surgery, LH/FSH ratio, fasting blood glucose, total cholesterol, triglyceride, high‐ and low‐density lipoprotein. Univariate and multivariate GAM were further fitted to account for the nonlinear relationships between Hb levels (g L^−1^) and PCOS and other indicators and phenotypes of PCOS.^[^ ^73^ ^]^ Nonlinear associations were visualized using the GAM partial effect plots, which demonstrated that how the smooth component of Hb concentrations in the model changed assuming that other variables were at their average value. A two‐tailed *P*‐value of less than 0.05 was considered statistically significant.

### Statistical Analysis—Mendelian Randomization and Bioinformatics Analyses

The multiplicative random effects IVW approach was employed as the main MR analysis to estimate the associations of genetically predicted Hb concentrations with PCOS and related traits. The IVW method provides the most accurate estimate under the assumption that all of the instruments are valid.^[^ ^74^ ^]^ Several sensitivity analyses, including MR‐Egger regression, weighted‐median method, and MR Pleiotropy RESidual Sum and Outlier test (MR‐PRESSO), were performed to explore the robustness of the results.^[^ ^75^, ^76^, ^77^ ^]^ MR‒Egger does not force the regression line through the intercept and is therefore able to detect the presence of directional pleiotropy.^[^ ^75^ ^]^ The weighted median provides robust causal effect estimates with the assumption that at least 50% of the SNPs used are valid.^[^ ^78^ ^]^ MR‐PRESSO was utilized to test the presence of outliers and correct pleiotropy via outlier removal.^[^ ^77^ ^]^ For exposure and outcome datasets with partial sample overlap, the type 1 error rate was additionally assessed to validate the causal associations.^[^ ^79^ ^]^ To confirm the validity of the directional assumption, the MR‐Steiger test was also performed. For binary outcomes, MR estimates were presented in terms of OR with 95% CIs, and for continuous outcomes, β coefficients with SE were used. the *F* statistic was computed to measure the strengths of IVs used in MR by the formula *F *= (*N *− *K *− 1/*K*)(*R* ^2^/1 − *R* ^2^), where *N* is the sample size and *K* is the number of IVs. *R* ^2^ was calculated as 2EAF(1 − EAF)*β* ^2^, where EAF is the effect allele frequency and *β* is the effect size of the effect allele of the IVs.

In the pathway enrichment analysis, the Benjamini‒Hochberg FDR correction was applied for multiple testing. In eQTL MR analysis, estimates were assessed using the Wald ratio method for instruments with one variant and the IVW method for instruments with two or more variants and the Bonferroni correction was used for the significance threshold.

A Bayesian statistical method was used to test colocalization of a shared causal genetic variant between protein levels and testosterone levels.^[^ ^80^ ^]^ The prior probabilities for genetic signal were set with default values, *P*1 = *P*2 = 1 × 10^−4^, *P*12 = 1 × 10^−5^, where *P*1 and *P*2 is the probability that a given genetic signal is associated with either of the two traits, and *P*12 is the probability associated with both traits. It computes the posterior probability of association for alternative hypotheses: H0, no association with either trait; H1, signal unique to trait 1; H2, signal unique to trait 2; H3, two distinct causal variants in the same locus, one for each trait; and H4, presence of a shared causal variant between two traits. It is strong evidence of colocalization if the PH4 value of 80% or higher.

### Statistical Analysis—In Vitro Validation Experiments

For the patients’ descriptive statistics, the Shapiro‐Wilk normality test was applied. Continuous variables that followed a normal distribution are represented as mean ± SD, while those did not follow a normal distribution are reported as median (interquartile range). If both groups displayed normal distributions and had equal variance, unpaired *t*‐test was used, otherwise nonparametric Mann‐Whitney *U*‐test was used.

### Statistical Analysis—Software Availability

Data manipulation, analyses, and visualization were performed using R version 4.2.1. In the observational study and in vitro experiments, the R packages “tidyverse” and “data.table” were used for data manipulation; “nnet” was used for multinomial logistic regression analysis; “mgcv” was used for GAM modelling; and “ggplot2” was used for data visualization. In the MR study, the R packages “TwoSampleMR” and “MR‐PRESSO” were used for two sample MR and MVMR analysis; type 1 error rate was obtained from an online calculator (https://sb452.shinyapps.io/overlap/); “coloc” was used for colocalization analysis; “clusterProfiler” was used for pathway enrichment; and “ggplot2” and “forestplot” were used for data visualization.

## Conflict of Interest

The authors declare no conflict of interest.

## Ethics Approval Statement

The ethics approval was obtained from the institutional review committee of the First Affiliated Hospital of Wenzhou Medical University (No. KY2022‐R096) and the Ethics Committee of Peking University Third Hospital (No. 2021‐533‐01). The ethical approvals and consent to participate involved in the MR section were available in the original GWAS study. The analyses were conducted in accordance with the approval and consent principles of the Helsinki Declaration of Ethics.

## Supporting information



Supporting Information

## Data Availability

All data generated or analyzed during this study are included in this published article and its supplementary information files.
