# Supplementary material for: Multiomics and Systematic Analyses Reveal the Roles of Hemoglobin and the HIF‐1 Pathway in Polycystic Ovary Syndrome
Source: Adv Sci (Weinh). 2025 Feb 14;12(14):2411679. doi: 10.1002/advs.202411679 (PMC11984896; doi:10.1002/advs.202411679)
Supplement: Supplementary file 1 — Supporting Information [file ADVS-12-2411679-s001.docx]

Supporting Information

Systematic analyses of the association between hemoglobin and polycystic ovary syndrome identify the potential effect of HIF-1 pathway

Guiquan Wang^1, Ϯ, *^, Weian Mao^2, Ϯ^, Yurong Zhang^3, Ϯ^, Haiyan Yang^4, Ϯ^, Ming Zhu^2^, Yan Li^4^, Wei Chen^5^, Yi Chen^2^, Chen Lou^2^, Ping Li^1^, Hsun-Ming Chang^6^, Shuai Yuan^7^, Yue Zhao^3, *^, Liangshan Mu^5, *^

^1^Department of Reproductive Medicine, Women and Children’s Hospital, School of Medicine, Xiamen University, Xiamen, China; Xiamen Key Laboratory of Reproduction and Genetics, Xiamen, China.

^2^The First School of Medicine, Wenzhou Medical University, Wenzhou, China.

^3^State Key Laboratory of Female Fertility Promotion, Center for Reproductive Medicine, Department of Obstetrics and Gynecology, Peking University Third Hospital; National Clinical Research Center for Obstetrics and Gynecology (Peking University Third Hospital); Key Laboratory of Assisted Reproduction, Ministry of Education; Beijing Key Laboratory of Reproductive Endocrinology and Assisted Reproductive Technology; Beijing, China.

^4^Reproductive Medicine Center, The First Affiliated Hospital of Wenzhou Medical University, Wenzhou, China.

^5^Reproductive Medicine Center, Zhongshan Hospital, Fudan University, Shanghai, China.

^6^Department of Obstetrics and Gynecology, China Medical University Hospital, Taichung, Taiwan.

^7^Unit of Cardiovascular and Nutritional Epidemiology, Institute of Environmental Medicine, Karolinska Institute, Stockholm, Sweden.

^Ϯ^The authors consider that the first four authors should be regarded as joint First Authors.

^*^These authors are corresponding authors.

**Contents**

**Figure S1.** Flow diagram of the eligibility criteria for the observational study.

**Table S1.** Baseline characteristics of the population in the observational study.

**Table S2.** Two sample Mendelian randomization results of Hb genetic instruments on PCOS and PCOS-related traits as outcomes.

**Table S3.** Type 1 error rate for the causal estimates for potential sample overlap.

**Table S4.** Reverse two-sample Mendelian randomization results of PCOS on Hb concentrations as outcome.

**Table S5.** Multivariable Mendelian randomization results for hemoglobin concentrations and testosterone on PCOS.

**Table S6.** The top 10 significant results for KEGG pathway enrichment analysis of candidate genes from Vuckovic et al. study’s Hb substantial cluster IVs with PCOS.

**Table S7.** The top 10 significant results for KEGG pathway enrichment analysis of candidate genes from Astle et al. study’s Hb substantial cluster IVs with PCOS.

**Table S8.** Significant two-sample Mendelian randomization results of HIF-1 pathway gene eQTL on PCOS and related traits as outcome.

**Table S9.** Colocalization analysis of corresponding pQTL and testosterone identified in eQTL MR analysis.

**Table S10.** Druggability of proteins potentially causally associated with PCOS.

**Table S11.** The clinical information of PCOS and control subjects for the gene expression analysis of blood leukocyte.

**Table S12.** The clinical information of PCOS and control subjects for the gene expression analysis of ovarian granulosa cells.

**Table S13.** The linear regression analysis between the hemoglobin levels and the candidate genes expression in the peripheral blood cells and granulosa cells.

**Table S14.** Description of GWAS details included in Mendelian randomization analysis.

**Table S15.** STROBE-MR checklist of Mendelian randomization studies.

**Figure S1.** Flow diagram of the eligibility criteria for the observational study.


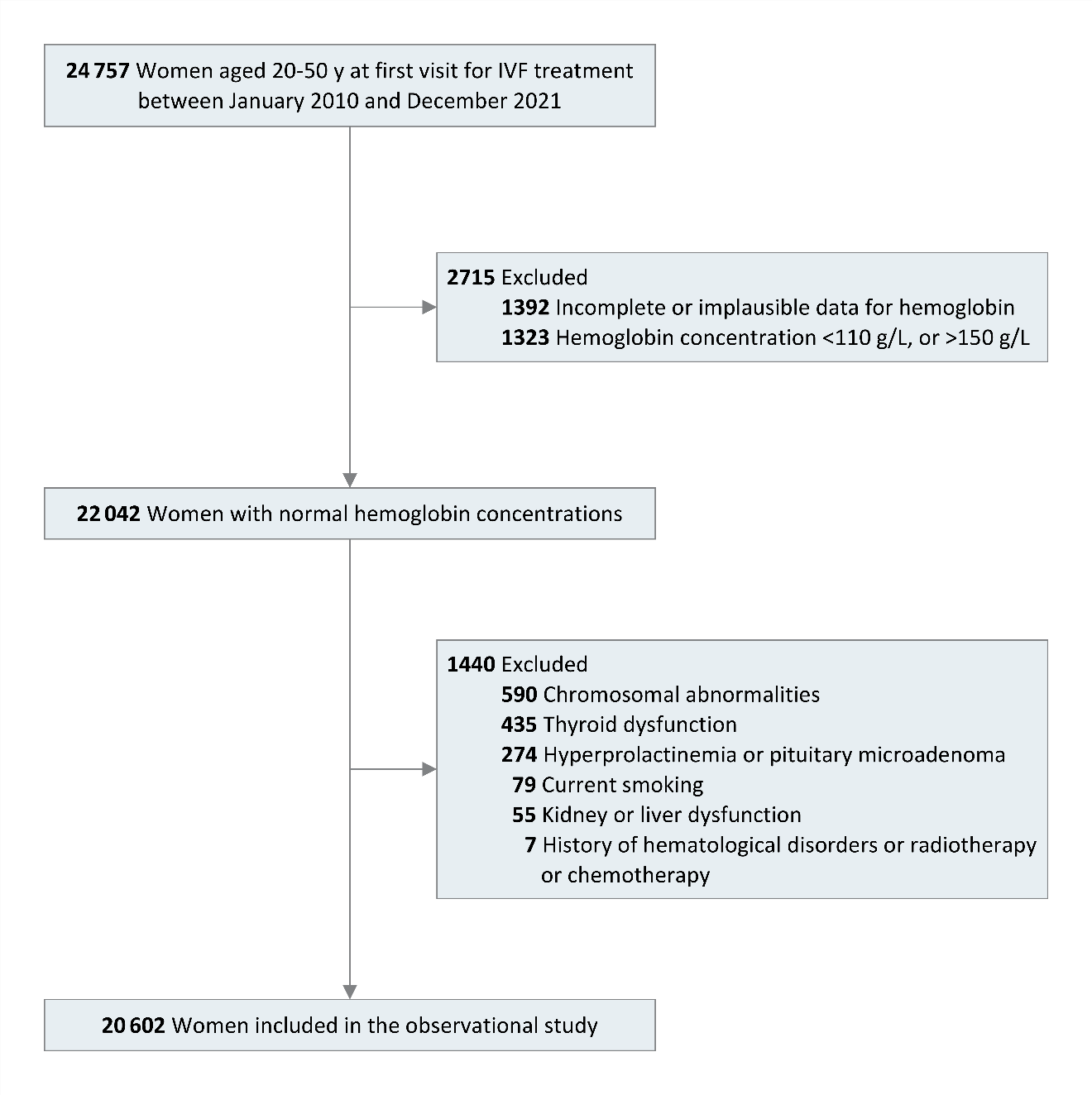


**Table S1.** Baseline characteristics of the population in the observational study.

| **Characteristics** | **Overall** | **Hemoglobin concentration tertiles** | | | ***P* value** |
| --- | --- | --- | --- | --- | --- |
|  |  | **110–130 g/L** | **131–138 g/L** | **139–150g/L** |  |
| Sample size | 20,602 | 6967 | 6884 | 6751 |  |
| Appointment date |  |  |  |  | <.001 |
| Between 2/3/2012 and 6/20/2014 | 3544 (17.2%) | 1301 (18.7%) | 1255 (18.2%) | 988 (14.6%) |  |
| Between 6/21/2014 and 8/12/2016 | 6484 (31.5%) | 2199 (31.6%) | 2194 (31.9%) | 2091 (31.0%) |  |
| Between 8/13/2016 and 12/24/2018 | 5898 (28.6%) | 2014 (28.9%) | 1965 (28.5%) | 1919 (28.4%) |  |
| Between 12/25/2018 and 12/31/2021 | 4676 (22.7%) | 1453 (20.9%) | 1470 (21.4%) | 1753 (26.0%) |  |
| Age, median (IQR), y | 31 (28–35) | 31 (28–35) | 31 (28–35) | 31 (28–34) | <.001 |
| Body mass index, median (IQR) | 21.4 (19.6–23.6) | 21.0 (19.5–23.0) | 21.4 (19.6–23.5) | 21.9 (19.9–24.4) | <.001 |
| Education, No. (%) |  |  |  |  | .03 |
| Primary or below | 1710 (8.4%) | 584 (8.4%) | 525 (7.7%) | 601 (9.0%) |  |
| Junior | 8682 (42.5%) | 2894 (41.8%) | 2937 (43.0%) | 2851 (42.6%) |  |
| High | 4003 (19.6%) | 1338 (19.3%) | 1314 (19.2%) | 1351 (20.2%) |  |
| Bachelor | 5899 (28.9%) | 2056 (29.7%) | 1994 (29.2%) | 1849 (27.6%) |  |
| Master or above | 143 (0.7%) | 46 (0.7%) | 58 (0.8%) | 39 (0.6%) |  |
| Systolic blood pressure, median (IQR), mmHg | 112 (104–121) | 111 (103–120) | 111 (103–120) | 114 (106–123) | <.001 |
| Diastolic blood pressure, median (IQR), mmHg | 72 (67–78) | 70 (65–77) | 71 (67–78) | 73 (68–80) | <.001 |
| Duration of infertility, median (IQR), y | 3 (2–5) | 3 (2–5) | 3 (2–5) | 3 (2–5) | .83 |
| Infertility type, No. (%) |  |  |  |  | .12 |
| Primary | 8680 (42.1%) | 2898 (41.6%) | 2884 (41.9%) | 2898 (42.9%) |  |
| Secondary | 11,922 (57.9%) | 4069 (58.4%) | 4000 (58.1%) | 3853 (57.1%) |  |
| Tubal factor, No. (%) |  |  |  |  | 0.233 |
| Yes | 14,257 (69.2%) | 4863 (69.8%) | 4777 (69.4%) | 4624 (68.5%) |  |
| No | 6345 (30.8%) | 2104 (30.2%) | 2107 (30.6%) | 2127 (31.5%) |  |
| Endometriosis, No. (%) |  |  |  |  | <.001 |
| Yes | 1958 (9.5%) | 732 (10.5%) | 677 (9.8%) | 549 (8.1%) |  |
| No | 18,644 (90.5%) | 6235 (89.5%) | 6207 (90.2%) | 6202 (91.9%) |  |
| History of ovarian surgery, No. (%) |  |  |  |  | .005 |
| Yes | 1352 (6.6%) | 482 (6.9%) | 484 (7.0%) | 386 (5.7%) |  |
| No | 19,250 (93.4%) | 6485 (93.1%) | 6400 (93.0%) | 6365 (94.3%) |  |
| Basal antral follicle count, median (IQR), n | 15 (10–21) | 14 (10–20) | 15 (10–21) | 16 (11–22) | <.001 |
| Basal follicle-stimulating hormone, median (IQR), mIU/mL | 7.5 (6.3–8.9) | 7.6 (6.4–9.1) | 7.5 (6.4–9.0) | 7.4 (6.2–8.8) | <.001 |
| Basal luteinizing hormone, median (IQR), mIU/mL | 4.6 (3.4–6.3) | 4.6 (3.5–6.2) | 4.6 (3.4–6.3) | 4.6 (3.3–6.4) | .94 |
| Ratio of basal luteinizing hormone to follicle-stimulating hormone, median (IQR) | 0.60 (0.43–0.84) | 0.59 (0.43–0.82) | 0.59 (0.43–0.84) | 0.61 (0.44–0.87) | <.001 |
| Basal estradiol, median (IQR), pmol/L | 152 (107–210) | 154 (106–212) | 152 (108–209) | 151 (107–208) | .18 |
| Basal testosterone, median (IQR), nmol/L | 1.4 (1.0–1.8) | 1.3 (0.9–1.6) | 1.4 (1.0–1.8) | 1.5 (1.1–1.9) | <.001 |
| Fasting blood glucose, median (IQR), mmol/L | 5.2 (4.9–5.5) | 5.1 (4.9–5.4) | 5.2 (5.0–5.5) | 5.2 (5.0–5.5) | <.001 |
| Total cholesterol, median (IQR), mmol/L | 4.5 (4.0–5.1) | 4.4 (3.9–4.9) | 4.5 (4.0–5.1) | 4.6 (4.1–5.2) | <.001 |
| Triglyceride, median (IQR), mmol/L | 0.9 (0.7–1.4) | 0.9 (0.7–1.2) | 0.9 (0.7–1.3) | 1.0 (0.8–1.5) | <.001 |
| High density lipoprotein, median (IQR), mmol/L | 1.4 (1.2–1.6) | 1.4 (1.2–1.6) | 1.4 (1.2–1.6) | 1.4 (1.2–1.6) | <.001 |
| Low density lipoprotein, median (IQR), mmol/L | 2.5 (2.1–3.0) | 2.4 (2.0–2.9) | 2.5 (2.1–3.0) | 2.6 (2.2–3.1) | <.001 |
| Ovulatory dysfunction, No. (%) |  |  |  |  | <.001 |
| Yes | 5198 (25.2%) | 1437 (20.6%) | 1671 (24.3%) | 2090 (31.0%) |  |
| No | 15,404 (74.8%) | 5530 (79.4%) | 5213 (75.7%) | 4661 (69.0%) |  |
| Polycystic ovarian morphology, No. (%) |  |  |  |  | <.001 |
| Yes | 5650 (27.4%) | 1640 (23.5%) | 1857 (27.0%) | 2153 (31.9%) |  |
| No | 14,952 (72.6%) | 5327 (76.5%) | 5027 (73.0%) | 4598 (68.1%) |  |
| Hyperandrogenism, No. (%) |  |  |  |  | <.001 |
| Yes | 3215 (15.6%) | 803 (11.5%) | 1053 (15.3%) | 1359 (20.1%) |  |
| No | 17,387 (84.4%) | 6164 (88.5%) | 5831 (84.7%) | 5392 (79.9%) |  |
| PCOS, No. (%) |  |  |  |  | <.001 |
| Yes | 3732 (18.1%) | 940 (13.5%) | 1207 (17.5%) | 1585 (23.5%) |  |
| No | 16,870 (81.9%) | 6027 (86.5%) | 5677 (82.5%) | 5166 (76.5%) |  |
| Phenotypes of PCOS, No. (%) |  |  |  |  | <.001 |
| A | 964 (4.7%) | 193 (2.8%) | 296 (4.3%) | 475 (7.0%) |  |
| B | 412 (2.0%) | 102 (1.5%) | 143 (2.1%) | 167 (2.5%) |  |
| C | 511 (2.5%) | 128 (1.8%) | 171 (2.5%) | 212 (3.1%) |  |
| D | 1845 (9.0%) | 517 (7.4%) | 597 (8.7%) | 731 (10.8%) |  |
| Non-PCOS | 16,870 (81.9%) | 6027 (86.5%) | 5677 (82.5%) | 5166 (76.5%) |  |

Abbreviations: IQR, interquartile range; PCOS, polycystic ovary syndrome.

**Table S2.** Two sample Mendelian randomization results of Hb genetic instruments on PCOS and PCOS-related traits as outcomes.

| **Stage** | **Outcome** | **Method** | **N** | **R^2^** | **F** | **MR effect ^*^** | ***P*** | ***P*_Heterogeneity_** | ***P*_Intercept_** | **MR-PRESSO Test** | **Outlier-corrected *P*** | **MR Steiger** |
| --- | --- | --- | --- | --- | --- | --- | --- | --- | --- | --- | --- | --- |
| Discovery ^†^ | PCOS | MR Egger | 445 | 0.08 | 110.8 | 1.725 (1.177, 2.526) | 0.005 | 0.002 | 0.066 | 0.002 | 0.001 | TRUE |
|  |  | IVW |  |  |  | 1.258 (1.048, 1.510) | 0.014 | 0.002 |  |  |  |  |
|  |  | Weighted median |  |  |  | 1.492 (1.137, 1.958) | 0.004 |  |  |  |  |  |
|  |  | Simple mode |  |  |  | 1.577 (0.737, 3.373) | 0.241 |  |  |  |  |  |
|  |  | Weighted mode |  |  |  | 1.766 (1.000, 3.120) | 0.051 |  |  |  |  |  |
|  | IM | MR Egger | 448 | 0.08 | 109.9 | 1.235 (0.809, 1.885) | 0.329 | 0.564 | 0.172 | 0.536 |  | TRUE |
|  |  | IVW |  |  |  | 0.953 (0.778, 1.169) | 0.646 | 0.552 |  |  |  |  |
|  |  | Weighted median |  |  |  | 0.914 (0.642, 1.302) | 0.619 |  |  |  |  |  |
|  |  | Simple mode |  |  |  | 0.717 (0.301, 1.705) | 0.452 |  |  |  |  |  |
|  |  | Weighted mode |  |  |  | 0.863 (0.490, 1.521) | 0.611 |  |  |  |  |  |
|  | PCO | MR Egger | 425 | 0.08 | 113.0 | 0.891 (0.307, 2.589) | 0.832 | 0.723 | 0.911 | 0.724 |  | TRUE |
|  |  | IVW |  |  |  | 0.940 (0.562, 1.573) | 0.814 | 0.734 |  |  |  |  |
|  |  | Weighted median |  |  |  | 0.940 (0.400, 2.214) | 0.888 |  |  |  |  |  |
|  |  | Simple mode |  |  |  | 0.298 (0.037, 2.387) | 0.255 |  |  |  |  |  |
|  |  | Weighted mode |  |  |  | 1.015 (0.325, 3.166) | 0.980 |  |  |  |  |  |
|  | BT | MR Egger | 460 | 0.08 | 112.2 | 0.025 (0.027) | 0.370 | 4.048×10^-169^ | 0.236 | <0.001 | 2.012×10^-8^ | TRUE |
|  |  | IVW |  |  |  | 0.053 (0.013) | 6.110×10^-5^ | 9.340×10^-170^ |  |  |  |  |
|  |  | Weighted median |  |  |  | 0.035 (0.013) | 0.008 |  |  |  |  |  |
|  |  | Simple mode |  |  |  | 0.035 (0.043) | 0.415 |  |  |  |  |  |
|  |  | Weighted mode |  |  |  | 0.011 (0.028) | 0.687 |  |  |  |  |  |
|  | TT | MR Egger | 460 | 0.08 | 112.2 | 0.033 (0.029) | 0.261 | 2.901×10^-140^ | 0.064 | <0.001 | 9.580×10^-11^ | TRUE |
|  |  | IVW |  |  |  | 0.079 (0.014) | 1.348×10^-8^ | 5.357×10^-142^ |  |  |  |  |
|  |  | Weighted median |  |  |  | 0.046 (0.015) | 0.002 |  |  |  |  |  |
|  |  | Simple mode |  |  |  | 0.010 (0.058) | 0.869 |  |  |  |  |  |
|  |  | Weighted mode |  |  |  | 0.028 (0.033) | 0.400 |  |  |  |  |  |
| Replication ^‡^ | PCOS | MR Egger | 119 | 0.05 | 81.9 | 1.120 (0.728, 1.721) | 0.608 | 0.011 | 0.219 | 0.007 | 1.131×10^-4^ | TRUE |
|  |  | IVW |  |  |  | 1.417 (1.144, 1.754) | 0.001 | 0.010 |  |  |  |  |
|  |  | Weighted median |  |  |  | 1.637 (1.211, 2.211) | 0.001 |  |  |  |  |  |
|  |  | Simple mode |  |  |  | 2.060 (0.979, 4.334) | 0.059 |  |  |  |  |  |
|  |  | Weighted mode |  |  |  | 1.685 (1.126, 2.523) | 0.013 |  |  |  |  |  |
|  | IM | MR Egger | 117 | 0.05 | 85.9 | 1.063 (0.671, 1.683) | 0.795 | 0.244 | 0.978 | 0.267 |  | TRUE |
|  |  | IVW |  |  |  | 1.057 (0.838, 1.333) | 0.639 | 0.265 |  |  |  |  |
|  |  | Weighted median |  |  |  | 1.194 (0.823, 1.733) | 0.350 |  |  |  |  |  |
|  |  | Simple mode |  |  |  | 0.475 (0.195, 1.154) | 0.103 |  |  |  |  |  |
|  |  | Weighted mode |  |  |  | 1.167 (0.656, 2.076) | 0.600 |  |  |  |  |  |
|  | PCO | MR Egger | 110 | 0.05 | 197.3 | 0.514 (0.166, 1.591) | 0.251 | 0.521 | 0.496 | 0.514 |  | TRUE |
|  |  | IVW |  |  |  | 0.721 (0.405, 1.284) | 0.267 | 0.536 |  |  |  |  |
|  |  | Weighted median |  |  |  | 0.959 (0.361, 2.548) | 0.934 |  |  |  |  |  |
|  |  | Simple mode |  |  |  | 0.328 (0.038, 2.866) | 0.316 |  |  |  |  |  |
|  |  | Weighted mode |  |  |  | 0.618 (0.191, 2.005) | 0.424 |  |  |  |  |  |
|  | BT | MR Egger | 126 | 0.06 | 83.4 | -0.017 (0.030) | 0.570 | 1.730×10^-52^ | 0.101 | <0.001 | 0.008 | TRUE |
|  |  | IVW |  |  |  | 0.026 (0.015) | 0.087 | 3.729×10^-54^ |  |  |  |  |
|  |  | Weighted median |  |  |  | 0.018 (0.015) | 0.217 |  |  |  |  |  |
|  |  | Simple mode |  |  |  | 0.090 (0.041) | 0.029 |  |  |  |  |  |
|  |  | Weighted mode |  |  |  | 0.010 (0.017) | 0.545 |  |  |  |  |  |
|  | TT | MR Egger | 126 | 0.06 | 83.4 | -0.016 (0.032) | 0.622 | 6.992×10^-45^ | 0.019 | <0.001 | 3.897×10^-4^ | TRUE |
|  |  | IVW |  |  |  | 0.050 (0.016) | 0.002 | 2.851×10^-48^ |  |  |  |  |
|  |  | Weighted median |  |  |  | 0.020 (0.016) | 0.203 |  |  |  |  |  |
|  |  | Simple mode |  |  |  | 0.041 (0.036) | 0.260 |  |  |  |  |  |
|  |  | Weighted mode |  |  |  | 0.021 (0.020) | 0.289 |  |  |  |  |  |

* For the binary variable outcome, the effect estimate is expressed as odds ratio (95% confidence interval), and for the continuous variable outcome, the effect is expressed as beta (standard error).

† IVs for hemoglobin concentration extracted from GWAS of Vuckovic et al. study published in Cell, 2020.

‡ IVs for hemoglobin concentration extracted from GWAS of Astle et al. study published in Cell, 2016.

Abbreviations: N, number of SNPs; F, F-statistic; *P*, *P*-value; Hb, hemoglobin; PCOS, polycystic ovary syndrome; IVW, inverse variance weighted; IM, irregular menstrual; PCO, polycystic ovaries; BT, bioavailable testosterone; TT, total testosterone; SHBG, sex hormone-binding globulin; SHBG adj, sex hormone-binding globulin adjusted for BMI.

**Table S3.** Type 1 error rate for the causal estimates for potential sample overlap.

| **Phenotype 1** | **Phenotype 2** | **N of Phenotype 1** | **N of Phenotype 2** | **Overlap proportion** | **N of IVs** | **R^2^** | **Estimate^*^** | **TypeⅠ error rate** |
| --- | --- | --- | --- | --- | --- | --- | --- | --- |
| Hb levels^†^ | Bioavailable testosterone | 173480 | 188507 | 23% | 126 | 0.06 | 0.026 | 0.05 |
| Hb levels^†^ | Total testosterone | 173480 | 230454 | 23% | 126 | 0.06 | 0.05 | 0.05 |
| Hb levels^‡^ | Bioavailable testosterone | 563085 | 188507 | 41% | 460 | 0.08 | 0.053 | 0.05 |
| Hb levels^‡^ | Total testosterone | 563085 | 230454 | 41% | 460 | 0.08 | 0.079 | 0.05 |

* For continuous outcome, estimates are effects of the change in the outcome in standard deviation units. For binary outcome, estimates are log odds ratios for the outcome per standard deviation change in the risk factor.

† IVs for hemoglobin concentration extracted from GWAS of Astle et al. study published in Cell, 2016.

‡ IVs for hemoglobin concentration extracted from GWAS of Vuckovic et al. study published in Cell, 2020.

Abbreviations: N, samplesize; IV, instrumental variable.

**Table S4.** Reverse two-sample Mendelian randomization results of PCOS on Hb concentrations as outcome.

| **Exposure** | **Outcome** | **Method** | **Discovery study ^*^** | | **Replication study ^†^** | |
| --- | --- | --- | --- | --- | --- | --- |
|  |  |  | **Beta (se)** | ***P*** | **Beta (se)** | ***P*** |
| Polycystic ovary syndrome | Hemoglobin | MR Egger | -0.048 (0.060) | 0.436 | -0.021 (0.060) | 0.736 |
|  |  | IVW | 0.001 (0.012) | 0.912 | 0.005 (0.012) | 0.692 |
|  |  | Weighted median | -0.018 (0.008) | 0.021 | -0.005 (0.014) | 0.705 |
|  |  | Simple mode | -0.024 (0.009) | 0.021 | -0.013 (0.024) | 0.593 |
|  |  | Weighted mode | -0.025 (0.009) | 0.017 | -0.017 (0.022) | 0.471 |

* IVs for hemoglobin concentration extracted from GWAS of Vuckovic et al. study published in Cell, 2020.

† IVs for hemoglobin concentration extracted from GWAS of Astle et al. study published in Cell, 2016.

**Table S5.** Multivariable Mendelian randomization results for hemoglobin concentrations and testosterone on PCOS.

| **Joint exposure** | **Exposure** | **Outcome** | **Discovery study ^*^** | | **Replication study ^†^** | |
| --- | --- | --- | --- | --- | --- | --- |
|  |  |  | **OR (95% CI)** | ***P*** | **OR (95% CI)** | ***P*** |
| Hb, BT | Hb | PCOS | 1.249 (1.046,1.491) | 0.014 | 1.359 (1.104, 1.675) | 0.004 |
|  | BT | PCOS | 1.745 (1.087, 2.800) | 0.021 | 1.570 (1.014, 2.431) | 0.043 |
| Hb, TT | Hb | PCOS | 1.232 (1.027, 1.479) | 0.025 | 1.293 (1.012,1.650) | 0.039 |
|  | BT | PCOS | 1.449 (0.849, 2.473 | 0.174 | 1.262 (0.736, 2.166) | 0.398 |

* IVs for hemoglobin concentration extracted from GWAS of Vuckovic et al. study published in Cell, 2020.

† IVs for hemoglobin concentration extracted from GWAS of Astle et al. study published in Cell, 2016.

Abbreviations: OR, odds ratio; CI, confidence interval; Hb, hemoglobin; BT, bioavailable testosterone; PCOS, polycystic ovary syndrome; TT, total testosterone.

**Table S6.** The top 10 significant results for KEGG pathway enrichment analysis of candidate genes from Vuckovic et al. study’s Hb substantial cluster IVs with PCOS.

| **KEGG ID** | **Description** | **Gene ratio ^*^** | ***P*** | **FDR** | **qvalue** |
| --- | --- | --- | --- | --- | --- |
| hsa04066 | HIF-1 signaling pathway | 11/173 | 8.66E-06 | 0.002478 | 0.002098 |
| hsa05202 | Transcriptional misregulation in cancer | 13/173 | 1.13E-04 | 0.016117 | 0.013643 |
| hsa04350 | TGF-beta signaling pathway | 9/173 | 2.75E-04 | 0.02624 | 0.022213 |
| hsa05216 | Thyroid cancer | 5/173 | 7.47E-04 | 0.052596 | 0.044524 |
| hsa01521 | EGFR tyrosine kinase inhibitor resistance | 7/173 | 9.20E-04 | 0.052596 | 0.044524 |
| hsa04929 | GnRH secretion | 6/173 | 1.60E-03 | 0.06752 | 0.057157 |
| hsa04022 | cGMP-PKG signaling pathway | 10/173 | 1.65E-03 | 0.06752 | 0.057157 |
| hsa04930 | Type II diabetes mellitus | 5/173 | 2.03E-03 | 0.069774 | 0.059065 |
| hsa05211 | Renal cell carcinoma | 6/173 | 2.35E-03 | 0.069774 | 0.059065 |
| hsa05230 | Central carbon metabolism in cancer | 6/173 | 2.53E-03 | 0.069774 | 0.059065 |

* Fraction whose numerator is the number of gene(s) enriched to the KEGG pathway and whose denominator is the number of all gene(s) entered for enrichment analysis.

Abbreviations: FDR, false discovery rate.

**Table S7.** The top 10 significant results for KEGG pathway enrichment analysis of candidate genes from Astle et al. study’s Hb substantial cluster IVs with PCOS.

| **KEGG ID** | **Description** | **Gene ratio ^*^** | ***P*** | **FDR** | **qvalue** |
| --- | --- | --- | --- | --- | --- |
| hsa04066 | HIF-1 signaling pathway | 5/55 | 0.000558 | 0.112101 | 0.097453 |
| hsa04930 | Type II diabetes mellitus | 3/55 | 0.002952 | 0.210965 | 0.1834 |
| hsa04666 | Fc gamma R-mediated phagocytosis | 4/55 | 0.003149 | 0.210965 | 0.1834 |
| hsa00603 | Glycosphingolipid biosynthesis - globo and isoglobo series | 2/55 | 0.004406 | 0.221384 | 0.192458 |
| hsa04670 | Leukocyte transendothelial migration | 4/55 | 0.005781 | 0.232389 | 0.202024 |
| hsa05202 | Transcriptional misregulation in cancer | 5/55 | 0.007081 | 0.234699 | 0.204033 |
| hsa05211 | Renal cell carcinoma | 3/55 | 0.009206 | 0.234699 | 0.204033 |
| hsa04650 | Natural killer cell mediated cytotoxicity | 4/55 | 0.009341 | 0.234699 | 0.204033 |
| hsa04910 | Insulin signaling pathway | 4/55 | 0.01061 | 0.23696 | 0.205998 |
| hsa00601 | Glycosphingolipid biosynthesis - lacto and neolacto series | 2/55 | 0.013225 | 0.242956 | 0.211211 |

* Fraction whose numerator is the number of gene(s) enriched to the KEGG pathway and whose denominator is the number of all gene(s) entered for enrichment analysis.

Abbreviations: FDR, false discovery rate.

**Table S8.** Significant two-sample Mendelian randomization results of HIF-1 pathway gene eQTL on PCOS and related traits as outcome.

| **Exposure** | **Outcome** | **Method** | **N** | **Beta** | **Se** | ***P*** | **Tissue** |
| --- | --- | --- | --- | --- | --- | --- | --- |
| LTBR | PCOS | Wald ratio | 1 | 0.211 | 0.101 | 3.743E-02 | Hypothalamus |
| LTBR | PCOS | Wald ratio | 1 | 0.127 | 0.061 | 3.743E-02 | Ovary |
| LTBR | PCOS | Wald ratio | 1 | 0.136 | 0.065 | 3.743E-02 | Pituitary |
| RPS6KB1 | PCOS | Wald ratio | 1 | 0.282 | 0.136 | 3.762E-02 | Pituitary |
| VHL | PCOS | Inverse variance weighted | 2 | -0.493 | 0.228 | 3.050E-02 | Whole blood |
| AKT1 | Bioavailable testosterone | Wald ratio | 1 | -0.033 | 0.011 | 4.316E-03 | Ovary |
| ARNT | Bioavailable testosterone | Wald ratio | 1 | 0.038 | 0.015 | 1.351E-02 | Pituitary |
| CAMK2B | Bioavailable testosterone | Inverse variance weighted | 3 | -0.024 | 0.008 | 1.884E-03 | Hypothalamus |
| ENO3 | Bioavailable testosterone | Wald ratio | 1 | 0.018 | 0.008 | 2.157E-02 | Pituitary |
| HK2 | Bioavailable testosterone | Inverse variance weighted | 5 | 0.034 | 0.017 | 3.734E-02 | Whole blood |
| INSR | Bioavailable testosterone | Wald ratio | 1 | 0.049 | 0.012 | 6.476E-05* | Whole blood |
| PDK1 | Bioavailable testosterone | Inverse variance weighted | 6 | 0.034 | 0.012 | 3.855E-03 | Whole blood |
| PFKL | Bioavailable testosterone | Inverse variance weighted | 2 | -0.018 | 0.009 | 4.110E-02 | Pituitary |
| PIK3CD | Bioavailable testosterone | Wald ratio | 1 | 0.114 | 0.056 | 4.265E-02 | Whole blood |
| PRKCA | Bioavailable testosterone | Inverse variance weighted | 3 | 0.058 | 0.012 | 2.028E-06* | Whole blood |
| RPS6KB1 | Bioavailable testosterone | Inverse variance weighted | 2 | -0.045 | 0.019 | 2.047E-02 | Whole blood |
| SLC2A4 | Bioavailable testosterone | Wald ratio | 1 | 0.024 | 0.009 | 9.471E-03 | Pituitary |
| ARNT | Total testosterone | Wald ratio | 1 | 0.030 | 0.010 | 3.695E-03 | Hypothalamus |
| HKDC1 | Total testosterone | Inverse variance weighted | 6 | -0.026 | 0.011 | 1.755E-02 | Whole blood |
| IFNGR2 | Total testosterone | Inverse variance weighted | 3 | 0.044 | 0.021 | 3.609E-02 | Whole blood |
| INSR | Total testosterone | Wald ratio | 1 | 0.047 | 0.014 | 5.431E-04 | Whole blood |
| MAP2K1 | Total testosterone | Inverse variance weighted | 2 | 0.079 | 0.031 | 1.060E-02 | Whole blood |
| MAP2K2 | Total testosterone | Wald ratio | 1 | 0.032 | 0.011 | 3.640E-03 | Hypothalamus |
| MAPK3 | Total testosterone | Inverse variance weighted | 13 | 0.036 | 0.014 | 8.649E-03 | Whole blood |
| MTOR | Total testosterone | Inverse variance weighted | 3 | 0.025 | 0.012 | 3.304E-02 | Whole blood |
| NFKB1 | Total testosterone | Wald ratio | 1 | 0.158 | 0.034 | 2.953E-06* | Whole blood |
| PRKCA | Total testosterone | Inverse variance weighted | 3 | 0.041 | 0.014 | 2.527E-03 | Whole blood |

This table presents the significant tissue-specified HIF-1 pathway eQTL MR associations on PCOS and related traits (*P* < 0.05 before correction for multiple testing).

*Significant tissue-specific eQTL MR results after Bonferroni correction.

Abbreviations: N, number of SNPs; *P*, *P*-value; PCOS, polycystic ovary syndrome; BT, bioavailable testosterone; TT, total testosterone.

**Table S9.** Colocalization analysis of corresponding pQTL and testosterone identified in eQTL MR analysis.

| **Gene** | **Traits** | **nsnps** | **H0** | **H1** | **H2** | **H3** | **H4** |
| --- | --- | --- | --- | --- | --- | --- | --- |
| NFKB1 | Total testerone | 2411 | 5.45E-14 | 1.85E-04 | 1.79E-11 | 5.97E-02 | 9.40E-01 |
| INSR | Bioavailable testosterone | 3649 | 5.00E-14 | 4.12E-04 | 1.21E-10 | 1.00E+00 | 3.14E-06 |
| PRKCA | Bioavailable testosterone | 5162 | 1.06E-23 | 2.73E-09 | 3.89E-15 | 1.00E+00 | 9.87E-09 |

**Table S10.** Druggability of proteins potentially causally associated with PCOS.

| **Gene** | **Protein** | **Drug or component name** | **Drug groups** | **Clinical trials of PCOS and associated conditions** | **Indications** | **Side effects** |
| --- | --- | --- | --- | --- | --- | --- |
| INSR | Insulin receptor | Insulin human | approved, investigational | PCOS / Insulin resistance | glycemic control in diabetes mellitus | hypoglycemia |
|  |  | Insulin lispro | approved | Endocrine System Diseases / Inflammation / Metabolic Diseases | glycemic control in diabetes mellitus | hypoglycemia |
|  |  | Linsitinib | investigational | Endocrine System Diseases | cancer/tumors (unspecified) and solid tumors | - |
| NFKB | Nuclear factor NF-kappa-B p105 subunit | HE3286 | investigational | Insulin resistance | inflammation, autoimmune diseases | - |
|  |  | Triflusal | approved, investigational | Insulin resistance | thromboembolic disorders / Stroke / myocardial infarction | higher risk of COVID-19 hospitalization and susceptibility to infection (PMID: 38191054) |
| PRKCA | Protein kinase C alpha type | Phosphatidyl serine | investigational, nutraceutical | Menstrual related disorders | cognitive impairment / Alzheimer's disease / age-associated memory impairment / non-Alzheimer's dementias | - |
|  |  | Ellagic acid | investigational | Metabolic syndrome | follicular lymphoma / brain injury in intrauterine growth restricted babies / obese adolescents / solar lentigines | - |
|  |  | alpha-Tocopherol succinate | approved, nutraceutical, vet_approved | Androgenic Alopecia | vitamin E deficiency | - |

**Table S11.** The clinical information of PCOS and control subjects for the gene expression analysis of blood leukocyte.

|  | **Control** | **PCOS** | ***P* value** |
| --- | --- | --- | --- |
| Number | 50 | 50 |  |
| Age, years | 31.40 ± 3.57 | 31.68 ± 3.99 | 0.712 |
| BMI, kg/m^2^ | 22.63 ± 2.91 | 23.81 ± 3.21 | 0.058 |
| Hemoglobin, g/L | 127.18 ± 10.60 | 139.46 ± 10.03 | **<0.001** |
| SBP, mmHg | 118.46 ± 8.85 | 120.77 ± 10.70 | 0.254 |
| DBP, mmHg | 73.38 ± 9.02 | 76.19 ± 8.94 | 0.130 |
| FSH, mIU/mL | 6.30 ± 2.21 | 6.00 ± 1.97 | 0.477 |
| LH, mIU/mL | 3.98 (2.95, 5.40) | 5.92 (4.18, 8.51) | **<0.001** |
| Estradiol, pmol/L | 140.38 ± 64.44 | 143.30 ± 64.88 | 0.825 |
| Total testosterone, nmol/L | 0.69 (069, 0.80) | 0.77 (0.69, 1.21) | **0.006** |
| Androstenedione, nmol/L | 4.69 (3.19, 6.70) | 8.96 (5.88, 11.00) | **<0.001** |
| AMH, nmol/L | 2.96 (2.00, 5.27) | 7.19 (4.49, 9.11) | **<0.001** |
| AFC | 13.00 (9.00, 17.00) | 24.00 (17.00, 24.00) | **<0.001** |
| FPG, mmol/L | 5.03 ± 0.40 | 5.04 ± 0.53 | 0.915 |
| TC, mmol/L | 4.57 ± 0.60 | 4.75 ± 0.68 | 0.194 |
| Triglycerides, mmol/L | 0.94 (0.71, 1.33) | 1.04 (0.79, 1.57) | 0.099 |
| HDL-C, mmol/L | 1.38 ± 0.27 | 1.30 ± 0.28 | 0.174 |
| LDL-C, mmol/L | 2.71 ± 0.51 | 2.92 ± 0.61 | 0.064 |

Abbreviations: FSH, follicle stimulating hormone; LH, luteinizing hormone; AMH, anti-Müllerian hormone; AFC, antral follicle counting; FPG, fasting plasma glucose; TC, total cholesterol; TG, triglycerides; LDL-C, low-density lipoprotein cholesterol; HDL-C, high-density lipoprotein cholesterol.

Mean ± standard deviation or median (interquartile range, IQR) are shown. The Mann–Whitney U test was used for non-normally distributed data, and the Student’s t test was used for normally distributed data.

**Table S12.** The clinical information of PCOS and control subjects for the gene expression analysis of ovarian granulosa cells.

|  | **Control** | **PCOS** | ***P* value** |
| --- | --- | --- | --- |
| Number | 50 | 50 |  |
| Age, years | 32.18 ± 3.99 | 32.00 ± 4.09 | 0.824 |
| BMI, kg/m^2^ | 22.57 ± 3.40 | 25.95 ± 4.76 | **<0.001** |
| Hemoglobin, g/L | 126.02 ± 8.41 | 137.44 ± 6.84 | **<0.001** |
| SBP, mmHg | 118.62 ± 13.96 | 127.44 ± 17.14 | **0.010** |
| DBP, mmHg | 74.38 ± 11.22 | 80.67 ± 11.97 | **0.013** |
| FSH, mIU/mL | 6.86 ± 2.16 | 6.27 ± 1.49 | **0.039** |
| LH, mIU/mL | 4.24 (3.08, 5.41) | 5.83 (3.55, 10.00) | **0.007** |
| Estradiol, pmol/L | 144.36 ± 80.85 | 158.38 ± 134.41 | 0.308 |
| Total testosterone, nmol/L | 0.69 (0.69, 0.72) | 1.18 (0.69, 1.63) | **<0.001** |
| Androstenedione, nmol/L | 4.31 (2.58, 6.48) | 9.84 (6.62, 13.70) | **<0.001** |
| AMH, nmol/L | 2.45 (1.65, 3.63) | 7.27 (5.06, 9.74) | **<0.001** |
| AFC | 13.00 (9.00, 17.00) | 24.00 (22.50, 30.75) | **<0.001** |
| FPG, mmol/L | 5.08 ± 0.46 | 5.15 ± 0.87 | 0.635 |
| TC, mmol/L | 4.87 ± 1.28 | 4.93 ± 1.02 | 0.806 |
| Triglycerides, mmol/L | 0.89 (0.67, 1.23) | 1.51 (0.89, 2.11) | **0.009** |
| HDL-C, mmol/L | 1.39 ± 0.30 | 1.18 ± 0.29 | **0.001** |
| LDL-C, mmol/L | 2.97 ± 0.94 | 2.89 ± 0.75 | 0.649 |

Abbreviations: FSH, follicle stimulating hormone; LH, luteinizing hormone; AMH, anti-Müllerian hormone; AFC, antral follicle counting; FPG, fasting plasma glucose; TC, total cholesterol; TG, triglycerides; LDL-C, low-density lipoprotein cholesterol; HDL-C, high-density lipoprotein cholesterol.

Mean ± standard deviation or median, interquartile range, IQR) are shown. The Mann–Whitney U test was used for non-normally distributed data, and the Student’s t test was used for normally distributed data.

**Table S13.** The linear regression analysis between the hemoglobin levels and the candidate genes expression in the peripheral blood cells and granulosa cells.

|  |  | **Unadjusted** | | **Adjusted with BMI** | |
| --- | --- | --- | --- | --- | --- |
|  |  | β [95% CI] | *P* value | β [95% CI] | *P* value |
| Peripheral blood cells | *INSR* | 4.451 (0.193, 8.709) | **0.041** | 4.434 (0.161, 8.707) | **0.042** |
|  | *NFKB1* | 3.449 (-2.124, 9.022) | 0.222 | 3.375 (-2.217, 8.967) | 0.234 |
|  | *PRKCA* | 1.858 (-3.735, 7.452) | 0.511 | 1.996 (-3.601, 7.594) | 0.48 |
| Granulosa cells | *INSR* | 4.729 (1.68, 7.780) | **0.003** | 5.272 (2.294, 8.250) | **0.001** |
|  | *NFKB1* | 1.416 (-0.200, 3.033) | 0.085 | 0.464 (-1.247, 2.175) | 0.592 |
|  | *PRKCA* | 2.161 (-1.399, 5.720) | 0.231 | 2.285 (-1.377, 5.947) | 0.218 |

**Table S14.** Description of GWAS details included in Mendelian randomization analysis.

| **Phenotype** | **Description** | **Contributing cohorts** | **Sex** | **Covariates** | **Inclusions/exclusions** | **Ancestry** | **Sample size** | **PMID/Link** |
| --- | --- | --- | --- | --- | --- | --- | --- | --- |
| Hemoglobin (Astle et al., 2016) | Concentration of hemoglobin with respect to unit of volume of blood | UK Biobank, UK BiLEVE, INTERVAL | Males and females | Technical covariables such as the time between venipuncture and full blood count (FBC) analysis, FBC instrument drift and calibration events and episodes of malfunction, non-technical sources of covariation such as age, sex and menopause status, BMI, smoking habits and alcohol consumption | UK Biobank exclusions: individuals suffering from blood cancers or other blood disorders. Participants who had a self-report or medical history containing a record of myelofibrosis, lymphoma, leukemia, malignant lymphoma, multiple myeloma, multiple myelofibrosis or myelodysplasia, chronic lymphocytic leukemia, chronic myeloid leukemia, acute myeloid leukemia, polycythemia vera, polycythemia, a myeloproliferative disorder, essential thrombocytosis, a hematological cancer histology report, an unspecified lymphatic or general hematological neoplasms, a myelodysplastic syndrome, or an unspecified heme malignancy, monoclonal gammopathy, an unspecified hereditary hematological disorder, hemochromatosis, thalassemia, hemophilia, sickle cell anemia, neutropenia, lymphopenia or pancytopenia. INTERVAL: cases of leukemia identified by screening the baseline FBC. | European | 173,480 | PMID: 27863252  https://www.ebi.ac.uk/gwas/publications/27863252 |
| Hemoglobin (Vuckovic et al., 2020) | Concentration of hemoglobin with respect to unit of volume of blood | Airwave, BioMe, CaPS,  CHS, Estonia, FHS, FINCAVAS, GERA, Health INTERVAL, MESA, MHI, MVP, RS, SHIP, UK Biobank, WHI, YFS | Males and females | Age, age-squared, sex, principal components and cohort specific covariates (e.g., study center, cohort, etc.) | Exclusions: pregnancy (when complete blood count (CBC) done), acute medical/surgical illness (when CBC done), blood cancer, leukemia, lymphoma, chemotherapy, myelodysplastic syndrome, bone marrow transplant, congenital or hereditary anemia (e.g., hemoglobinopathy such as sickle cell anemia or thalassemia), HIV, end-stage kidney disease, dialysis, EPO treatment, splenectomy, cirrhosis and those with any of the following extreme measurements: WBC count > 100*109/L with > 5% immature cell or blasts, WBC > 200*109/L, Hemoglobin > 20 g/dL, Hematocrit > 60%, Platelet > 1000*109/L. | European | 563,085 | PMID: 32888494  http://www.mhi-humangenetics.org/en/resources/ |
| PCOS | PCOS diagnosis was based on NIH criteria or Rotterdam criteria. | Rotterdam, UK (London/ Oxford), EGCUT, deCODE, Chicago, Boston | Females | Age, BMI. | Boston: Controls were screened for regular menses and no hyperandrogenism. | European | 10,074 cases/  103,164 controls | PMID: 30566500  https://doi.org/10.17863/CAM.27720 |
| Total testosterone in women | Testosterone (UK Biobank variable 30850) including people where testosterone was not reportable as below lower limit (UK Biobank variable 30856=4). | UK Biobank | Females | Fasting time, age, center, chip/release of genetic data. | Participants with missing values that were not reportable at assay as too low were set to 0.3, a value below the lower limit of detection. Included white Europeans only. | European | 230,454 | PMID: 32042192  https://www.ebi.ac.uk/gwas/publications/32042192 |
| Bioavailable testosterone in women | Bioavailable testosterone, calculated from testosterone, accounting for concentration of SHBG and albumin using Vermeulen equation. | UK Biobank | Females | Age, dilution, batch, mins since blood draw, time of blood draw, menopause, operation status. Includes individuals at lower limit. | Women who self-reported taking hormone-based medication including HRT and oral contraception at the time of the initial visit (UK Biobank variables 30850 and 20003). Included white Europeans only. | European | 188,507 | PMID: 32042192  https://www.ebi.ac.uk/gwas/publications/32042192 |
| Polycystic ovaries | ICD 10: E28.2 | UK Biobank | Females | Genetic relatedness, sex, birth year and the first 4 principal components | Not available. | European | 209 cases/  405,386 controls | https://pheweb.org/UKB-SAIGE/phenotypes |
| Irregular menstrual | ICD 10: N92.6 | UK Biobank | Females | Genetic relatedness, sex, birth year and the first 4 principal components | Not available. | European | 1,989 cases/  377,857 controls | https://pheweb.org/UKB-SAIGE/phenotypes |
| Plasma protein | Plasma protein levels on Aptamer-based (SOMAmer) technology | deCODE genetics | Males and females | Age, sex and sample age. | Not available. | European | 35,559 | PMID: 34857953  https://www.decode.com/summarydata/ |

**Table S15.** STROBE-MR checklist of Mendelian randomization studies.

| **Item No.** | **Section** | **Checklist item** | **Relevant text from manuscript** | |
| --- | --- | --- | --- | --- |
| 1 | **TITLE and ABSTRACT** | Indicate Mendelian randomization (MR) as the study’s design in the title and/or the abstract if that is a main purpose of the study | "Mendelian randomization" is named in the abstract. | |
|  | **INTRODUCTION** |  |  |  |
| 2 | **Background** | Explain the scientific background and rationale for the reported study. What is the exposure? Is a potential causal relationship between exposure and outcome plausible? Justify why MR is a helpful method to address the study question | In the Introduction (paragraph 2), we introduce why hemoglobin concentration is a plausible causal exposure for polycystic ovary syndrome. In the paragraph 3 of Introduction section, we introduce the rationale for using MR to explore the causal question. | |
| 3 | **Objectives** | State specific objectives clearly, including pre-specified causal hypotheses (if any). State that MR is a method that, under specific assumptions, intends to estimate causal effects | See Introduction (paragraph 3 and 4). | |
|  | **METHODS** |  |  |  |
| 4 | **Study design and data sources** | Present key elements of the study design early in the article. Consider including a table listing sources of data for all phases of the study. For each data source contributing to the analysis, describe the following: |  | |
|  | a) | Setting: Describe the study design and the underlying population, if possible. Describe the setting, locations, and relevant dates, including periods of recruitment, exposure, follow-up, and data collection, when available. | Available information about the GWAS studies is provided in the Table S14. Further information is given in the original GWAS publications referenced. | |
|  | b) | Participants: Give the eligibility criteria, and the sources and methods of selection of participants. Report the sample size, and whether any power or sample size calculations were carried out prior to the main analysis | See Table S14. | |
|  | c) | Describe measurement, quality control and selection of genetic variants | See Table S14. Age, sex and principal components were used as covariates. | |
|  | d) | For each exposure, outcome, and other relevant variables, describe methods of assessment and diagnostic criteria for diseases | See Table S14. | |
|  | e) | Provide details of ethics committee approval and participant informed consent, if relevant | The GWAS summary data used in this study are all publicly available and described in the Table S14. For more information on ethics committee approval and participant informed consent, please refer to the original GWAS publication. | |
| 5 | **Assumptions** | Explicitly state the three core IV assumptions for the main analysis (relevance, independence and exclusion restriction) as well assumptions for any additional or sensitivity analysis | Assumptions introduced in the introduction and then described in relation to analyses in the “Mendelian randomization analyses” section of methods. | |
| 6 | **Statistical methods: main analysis** | Describe statistical methods and statistics used |  | |
|  | a) | Describe how quantitative variables were handled in the analyses (i.e., scale, units, model) | See “Mendelian randomization and bioinformatics analyses” section of methods. | |
|  | b) | Describe how genetic variants were handled in the analyses and, if applicable, how their weights were selected | See “Mendelian randomization analyses” and “Pathway enrichment analyses and pathway-specific Mendelian randomization analysis” section of methods. | |
|  | c) | Describe the MR estimator (e.g. two-stage least squares, Wald ratio) and related statistics. Detail the included covariates and, in case of two-sample MR, whether the same covariate set was used for adjustment in the two samples | See “Mendelian randomization and bioinformatics analyses” section of methods. Included covariates are shown in the Table S14. | |
|  | d) | Explain how missing data were addressed | See “Pathway enrichment analyses and pathway-specific Mendelian randomization analysis” section of methods. | |
|  | e) | If applicable, indicate how multiple testing was addressed | See “Mendelian randomization and bioinformatics analyses” section of methods. | |
| 7 | **Assessment of assumptions** | Describe any methods or prior knowledge used to assess the assumptions or justify their validity | See “Mendelian randomization and bioinformatics analyses” section of methods. | |
| 8 | **Sensitivity analyses and additional analyses** | Describe any sensitivity analyses or additional analyses performed (e.g. comparison of effect estimates from different approaches, independent replication, bias analytic techniques, validation of instruments, simulations) | See “Mendelian randomization and bioinformatics analyses” section of methods. | |
| 9 | **Software and pre-registration** |  |  |  |
|  | a) | Name statistical software and package(s), including version and settings used | See the “Software availability” section of methods. | |
|  | b) | State whether the study protocol and details were pre-registered (as well as when and where) | The study protocol was not pre-registered. | |
|  | **RESULTS** |  |  |  |
| 10 | **Descriptive data** |  |  |  |
|  | a) | Report the numbers of individuals at each stage of included studies and reasons for exclusion. Consider use of a flow diagram | See Table S14. | |
|  | b) | Report summary statistics for phenotypic exposure(s), outcome(s), and other relevant variables (e.g. means, SDs, proportions) | See Table S14. | |
|  | c) | If the data sources include meta-analyses of previous studies, provide the assessments of heterogeneity across these studies | See Table S14. For the assessments of heterogeneity of GWAS meta-analyses, please refer to the original GWAS publication. | |
|  | d) | For two-sample MR:  i.  Provide justification of the similarity of the genetic variant-exposure associations between the exposure and outcome samples  ii.  Provide information on the number of individuals who overlap between the exposure and outcome studies | See Table S14. | |
| 11 | **Main results** |  |  |  |
|  | a) | Report the associations between genetic variant and exposure, and between genetic variant and outcome, preferably on an interpretable scale | See “MR analyses revealed the causal association of Hb levels with PCOS and testosterone” section of results and Figure 3. | |
|  | b) | Report MR estimates of the relationship between exposure and outcome, and the measures of uncertainty from the MR analysis, on an interpretable scale, such as odds ratio or relative risk per SD difference | See “MR analyses revealed the causal association of Hb levels with PCOS and testosterone” and “Key genes were identified using eQTL and pQTL MR analyses”s section of results and Figure 3. | |
|  | c) | If relevant, consider translating estimates of relative risk into absolute risk for a meaningful time period | Not applicable. | |
|  | d) | Consider plots to visualize results (e.g. forest plot, scatterplot of associations between genetic variants and outcome versus between genetic variants and exposure) | See Figure 3. | |
| 12 | **Assessment of assumptions** |  |  |  |
|  | a) | Report the assessment of the validity of the assumptions | See Table S2-8 and text results. | |
|  | b) | Report any additional statistics (e.g., assessments of heterogeneity across genetic variants, such as *I^2^*, Q statistic or E-value) | See Table S2. | |
| 13 | **Sensitivity analyses and additional analyses** |  |  |  |
|  | a) | Report any sensitivity analyses to assess the robustness of the main results to violations of the assumptions | See “MR analyses revealed the causal association of Hb levels with PCOS and testosterone” section of results and Figure 3. | |
|  | b) | Report results from other sensitivity analyses or additional analyses | See comment above. | |
|  | c) | Report any assessment of direction of causal relationship (e.g., bidirectional MR) | See “MR analyses revealed the causal association of Hb levels with PCOS and testosterone” section of results and Table S3. | |
|  | d) | When relevant, report and compare with estimates from non-MR analyses | Estimates from both observational studies, MR analyses, and in tiro experiments are shown in the results section. | |
|  | e) | Consider additional plots to visualize results (e.g., leave-one-out analyses) | See Figure 3. | |
|  | **DISCUSSION** |  |  |  |
| 14 | **Key results** | Summarize key results with reference to study objectives | See Discussion paragraph 1. | |
| 15 | **Limitations** | Discuss limitations of the study, taking into account the validity of the IV assumptions, other sources of potential bias, and imprecision. Discuss both direction and magnitude of any potential bias and any efforts to address them | See “Limitations” section. | |
| 16 | **Interpretation** |  |  |  |
|  | a) | Meaning: Give a cautious overall interpretation of results in the context of their limitations and in comparison with other studies | See Discussion paragraph 2-5 | |
|  | b) | Mechanism: Discuss underlying biological mechanisms that could drive a potential causal relationship between the investigated exposure and the outcome, and whether the gene-environment equivalence assumption is reasonable. Use causal language carefully, clarifying that IV estimates may provide causal effects only under certain assumptions | See Discussion paragraph 3-5. | |
|  | c) | Clinical relevance: Discuss whether the results have clinical or public policy relevance, and to what extent they inform effect sizes of possible interventions | Done throughout the discussion. | |
| 17 | **Generalizability** | Discuss the generalizability of the study results (a) to other populations, (b) across other exposure periods/timings, and (c) across other levels of exposure | See “Limitations” section. | |
|  | **OTHER INFORMATION** |  |  |  |
| 18 | **Funding** | Describe sources of funding and the role of funders in the present study and, if applicable, sources of funding for the databases and original study or studies on which the present study is based | Funding requirements completed in accordance with journal guidelines. | |
| 19 | **Data and data sharing** | Provide the data used to perform all analyses or report where and how the data can be accessed, and reference these sources in the article. Provide the statistical code needed to reproduce the results in the article, or report whether the code is publicly accessible and if so, where | Data and data sharing requirements completed in accordance with journal guidelines. | |
| 20 | **Conflicts of Interest** | All authors should declare all potential conflicts of interest | All authors declare no conflicts of interest. | |
